# High-resolution single-cell RNA sequencing using canFam4 reveals novel immune subsets and checkpoint programs in healthy dogs

**DOI:** 10.3389/fimmu.2025.1680437

**Published:** 2025-12-18

**Authors:** Myung-Chul Kim, Taeeun Gu, Hyeewon Seo, Yewon Moon, Nicholas Borcherding, Ryan Kolb, Yubin Kim, Youngmin Yun, Woo-Jin Song, Chung-Young Lee, Hyun Je Kim, Weizhou Zhang

**Affiliations:** 1 Laboratory of Clinical Pathology, College of Veterinary Medicine, Kyungpook National University, Daegu, Republic of Korea; 2 Department of Biomedical Sciences, Seoul National University Graduate School, Seoul, Republic of Korea; 3 Department of Pathology & Immunology, Washington University School of Medicine in St. Louis, St. Louis, MO, United States; 4 Department of Pathology, Immunology and Laboratory Medicine, University of Florida College of Medicine, Gainesville, FL, United States; 5 College of Veterinary Medicine, Jeju National University, Jeju, Republic of Korea; 6 Laboratory of Veterinary Internal Medicine, College of Veterinary Medicine, Jeju National University, Jeju, Republic of Korea; 7 Signiture Animal Medical Center, Seoul, Republic of Korea; 8 Department of Microbiology, School of Medicine, Kyungpook National University, Daegu, Republic of Korea; 9 Untreatable Infectious Disease Institute, Kyungpook National University, Daegu, Republic of Korea; 10 Genomic Medicine Institute, Seoul National University Medical Research Center, Seoul, Republic of Korea; 11 Department of Microbiology and Immunology, Seoul National University College of Medicine, Seoul, Republic of Korea; 12 Cancer Research Institute, Seoul National University College of Medicine, Seoul, Republic of Korea; 13 UF Health Cancer Center, University of Florida, Gainesville, FL, United States

**Keywords:** canFam4, circulating leukocytes, reference transcriptome, ScRNA-seq, translational model

## Abstract

**Introduction:**

Single-cell RNA sequencing (scRNA-seq) enables high-resolution profiling of immune heterogeneity. Although previous studies have mapped the single-cell transcriptomic atlases of peripheral leukocytes in healthy dogs, the identification and functional characterization of distinct immune subsets remain incomplete.

**Methods:**

We constructed a single-cell atlas of peripheral leukocytes from six healthy small-breed dogs using the 10x Genomics platform and the updated canFam4 genome.

**Results and discussion:**

Analysis of 30,040 high-quality transcriptomes revealed 51 distinct immune subsets, including *CD14*^+^*CD33*^+^ monocytes, *XCR1*^+^*CD1D*^+^ dendritic cells, *CEACAM1*^+^*CD24*^+^ neutrophils, and *IL32*^+^*BATF*^+^ regulatory T cells, which were underrepresented in canFam3.1-based studies. Interferon-enriched *CD14*^+^ monocytes and *CD4*^+^ T subsets associated with myxomatous mitral valve disease were also identified. Functional enrichment analyses suggested that *PDCD1* is associated with attenuated TCR signaling, whereas *LAG3* was associated with malate metabolism pathways in *CD4*^+^ T cells and reduced *TBX21* expression in *CD8*^+^ T cells linked to antiviral responses. *CD274*, which encodes PD-L1 was linked to IL-10 production in neutrophils, and *CTLA4* represented an initial activation of double-negative T subsets. T cell exhaustion scores and proliferative fractions varied across cohorts, reflecting differences in environmental antigenic exposures.

**Conclusion:**

To our knowledge, this study represents the first comprehensive, gene-resolved single-cell analysis to reveal immunoregulatory checkpoint mechanisms underlying immune homeostasis in healthy dogs. Our dataset will serve as a valuable resource for future comparative and translational immunology research in dogs.

## Introduction

Humans and dogs inextricably share not only genetic traits but also environments, lifestyles, stressors, and microbial exposures (1, 2). Moreover, dogs have many of the same naturally occurring diseases as humans (3), including cancer, inflammatory bowel disease, and cardiomyopathy (2, 4). Consequently, companion dogs serve as valuable spontaneous models for translating basic science into clinical applications (5); however, cross-fertilization between veterinary and human medicine is most advanced in oncology (6).

Single-cell RNA sequencing (scRNA-seq) dissects dynamic and heterogeneous tissue microenvironments by characterizing the transcriptome at the single-cell level, thereby improving our understanding of cell identity, fate, and function in the context of both normal biology and pathology (7). To date, scRNA-seq has begun to unlock the secrets of veterinary diseases with application to canine cells derived from blood (8–13), lymphoid tissues (14–16), bronchoalveolar lavage (17), hippocampus (18), liver (16), lung (19), adipose tissues (20), inflamed tissues (12, 21–23), and cancers (10, 24–27). More recently, the single-cell transcriptome atlas has elucidated canine hematopoiesis (28) and pathogenesis of peri-implantitis via pseudotime and interactome analyses (29). Among various sample origins, peripheral blood mononuclear cells (PBMCs) have served as an easily accessible and valuable source for gaining insight into the tumor microenvironment (8, 13), lymphocyte clonal expansion (10), inflammatory disease etiology (23), innate immunity (16), aging (20), and preclinical immunotherapy optimization (12).

Although the canine genome has been sequenced, the commonly used reference genome (canFam3.1) remains incomplete (30), with gaps in transcript annotation, the absence of key immune-related genes, e.g. *CD14*, *FCGR3A*, CD1 family, and a portion of scRNA-seq reads that remain unmapped due to deficient or partial genomic regions (8–12). Recently, new canine genome references, such as GSD_1.0 (canFam4), have been released, offering potential for improved transcriptional resolution with high contiguity (30). Therefore, evaluating canFam4 in the context of scRNA-seq analysis may enhance single-cell recovery and improve immune cell-type identification, yet this has not been systematically examined to date.

Prior studies have profiled canine leukocytes using scRNA-seq (8–10, 23). However, the identification and functional characterization of distinct immune subsets remain incomplete, and it is unclear whether all clinically relevant cell states have been fully captured. To address these gaps, we performed scRNA-seq on PBMCs from clinicopathologically healthy, client-owned, indoor small-breed dogs. To our knowledge, this study demonstrates, for the first time, that canFam4 substantially increases cell recovery and enables the detection of previously unannotated markers, such as *CD14*. In addition, we provide immune subsets potentially involved in disease and regulatory mechanisms of immune checkpoint genes, such as *PDCD1*, *CTLA4*, *LAG3*, and *CD274* in dogs. To our knowledge, this is the first scRNA-seq study to provide molecular evidence of how canine immune subsets maintain homeostasis. Our dataset and methodology establish a foundational resource for future investigations into canine cancers and other immune-related disorders.

## Materials and methods

### Study subject enrollment and inclusion criteria

Client-owned adult to geriatric dogs (7 to 12 years old) were enrolled from the Veterinary Medical Teaching Hospital at Jeju National University (Jeju-si, South Korea), comprising two recognized breeds, Maltese (n = 2) and Poodle (n = 1), as well as mixed-breed dogs (n = 3). All dogs were housed indoors and confirmed by their owners to have no preexisting disease conditions. Inclusion criteria included absence of clinical signs of disease on physical examination, normal clinicopathologic results, and no vaccinations or treatments within four weeks before blood sampling. Written informed consent was obtained from each owner before enrollment. The study protocol was reviewed and approved by the Jeju National University Institutional Animal Care and Use Committee (IACUC No. 20230072).

### Clinicopathologic examinations

Resident veterinarians conducted thorough physical examinations and clinical assessments. Hematological and biochemical analyses were performed using the ProCyte Dx and Catalyst One systems (IDEXX Laboratories, MA, USA), respectively. To screen for Babesia infection, we employed both a point-of-care antibody rapid test (Canine Babesia Antibody Rapid Kit, BioNote Inc., Gyeonggi-do, South Korea) and real-time PCR (CareDx™ Canine Babesia Real-Time PCR Kit, Carevet Inc., Gyeonggi-do, South Korea). Additionally, Anigen Rapid CaniV-4 (BioNote Inc.) and SNAP 4Dx Plus (IDEXX Laboratories) assays were used to detect other protozoal pathogens. Only dogs testing negative for all infectious agents were included in the study, and six healthy dogs were selected for study.

### Isolation of peripheral blood mononuclear cells

Blood was collected via jugular venipuncture and processed immediately. PBMCs were isolated by density gradient centrifugation using SepMate-15 tubes (Stemcell Technologies, Vancouver, Canada) and Ficoll-Paque PLUS (GE Healthcare, Chicago, IL, USA). The PBMC fraction was washed twice with Dulbecco’s phosphate-buffered saline (DPBS; Thermo Fisher Scientific, Waltham, MA, USA) containing 2% heat-inactivated fetal bovine serum (FBS; Life Technologies, Pleasanton, CA, USA). A small aliquot of the suspension was cytospun onto glass slides and stained with Diff-Quik to confirm the purity and composition of isolated cells. The remaining cells were cryopreserved in a cytoprotective medium (Cellbanker 1, Zenogen Pharma, Koriyama, Japan) and stored in liquid nitrogen for further analysis.

### Fluorescence-activated cell sorting

Thawed PBMCs were washed and incubated with an anti-dog Fc receptor blocking reagent (Invitrogen, eBioscience, San Diego, CA, USA) on ice for 10 minutes. After washing with ice-cold DPBS containing 2% FBS, cells were stained for 30 minutes at 37 °C, protected from light, with Fixable Viability Dye eFluor 780 (Invitrogen, eBioscience), anti-dog CD45 PE (clone YKIX 716.13; Bio-Rad Laboratories, Hercules, CA, USA), and anti-dog CD3 FITC (clone CA17.2A12; Bio-Rad Laboratories). Live single cells were then sorted on a FACSAria III (BD Biosciences Pharmingen, San Diego, CA, USA), gating on CD45^+^CD3^+^ T cells and CD45^+^CD3^-^ non-T leukocytes. Post-sort viability and cell counts were assessed using a LUNA-FL™ Automated Fluorescence Cell Counter (Logos Biosystems, Anyang, South Korea). Equal numbers of CD45^+^CD3^+^ and CD45^+^CD3^-^ cells from each separate dog were pooled within the same animal into a new tube, which immediately processed for single-cell library construction on the 10x Genomics Chromium platform in a single run.

### 10X genomics single-cell RNA sequencing library construction

Single-cell libraries were prepared using the Chromium Next GEM Single Cell 5′ Kit v2 (10x Genomics) following the manufacturer’s protocol (31, 32). Libraries were sequenced on an Illumina NovaSeq 6000 with 2 × 150 bp paired-end reads. Base calling and FASTQ generation were performed with bcl2fastq (Illumina) and cellranger mkfastq (Cell Ranger v8.0.0, 10x Genomics). Reads were aligned and quantified using cellranger count by using two canine genome references: CanFam3.1 (GCA_000002285.3) and GSD_1.0/CanFam4 (GCF_011100685.1). Genome references were constructed with cellranger mkref using filtered FASTA and GTF files containing only protein-coding genes.

### single-cell RNA sequencing data integration, initial preprocessing, and sub-clustering

All single-cell data were processed and analyzed in Seurat v5.1.0 (R v4.2). After loading each sample, low-quality cells were filtered out if they met any of the following criteria: fewer than 200 or more than 4000 unique features and a mitochondrial gene content of 10%. Filtered datasets were normalized using Seurat’s default workflow. Next, all six samples were merged and integrated into a single Seurat object using 3,000 anchor features. Principal component analysis (PCA) was performed for dimensionality reduction, and principal components (PCs) 1–30, which were selected based on JackStraw significance, *p*-value < 1e-5, and percentage variance explained, were used for downstream clustering. We applied graph-based clustering on both t-distributed Stochastic Neighbor Embedding (t-SNE) and Uniform Manifold Approximation and Projection (UMAP) embeddings, using a final resolution of 3.7 that was empirically determined through iterative evaluation of cluster separability to preserve biologically meaningful immune subsets. The simultaneous use of UMAP and t-SNE follows previous immune profiling strategy that combined both algorithms to provide complementary views of global and subset-level cellular structures (33). To determine cohort-specific phenotypes or validate our findings, we incorporated publicly available canine scRNA-seq datasets, including peripheral blood TCR αβ T cells (GSE218355) (9) and PBMCs (GSE225599) (8). FASTQ files from GSE144730—canine PBMCs in atopic dermatitis (23)—were not included due to unsupported library chemistry. Available external datasets were processed and integrated alongside our own using the same quality control, normalization, and anchor-based integration pipeline. Harmony integration (v1.2.0) was performed on the PCA-reduced data to correct batch effects across studies. The first 30 Harmony dimensions were subsequently used for neighbor finding, clustering, UMAP, and t-SNE visualization. Doublets were detected and removed with scDblFinder v1.18.0 (31). Single-cell clusters were annotated by canonical lineage markers, published signatures for rare populations, and unbiased cell-type inference via SingleR v2.6.0 (34) using celldex v1.14.0 reference panels. Finally, the escape v2.0.0 package (35) was used to calculate enrichment scores for specific immune gene signatures derived from original canine studies (8, 22). Additionally, scRNA-seq datasets GSE225599 and GSE252470, generated from circulating leukocytes and tumor-infiltrating immune cells in canine osteosarcoma, were analyzed independently as an external cohort to validate our findings.

### Differentially expressed gene analysis

Differential expression was assessed using Seurat’s likelihood-ratio test, comparing each cluster to all other cells. Marker genes for individual clusters were identified with FindAllMarkers, applying cutoffs of an absolute log_2_-fold change > 0.25 and ≥ 25% of cells in the cluster expressing the gene. To compare cluster markers and differentially expressed genes (DEGs) across experimental groups, we used FindMarkers with a more stringent threshold (absolute log_2_ fold change > 0.5 and *p*< 0.05).

### Gene set enrichment analysis and gene ontology analysis

We performed gene set enrichment analysis (GSEA) using the escape R package, employing Hallmark gene sets from the Molecular Signatures Database and those from previous canine studies (36–40). DEGs were further analyzed for Gene Ontology (GO) enrichment using both the PANTHER annotation (for *Canis lupus familiaris*) and the ShinyGO v0.80 web tool with species set to dog. GO terms were considered significant at *p* < 0.05 and false discovery rate (FDR) < 0.05. Pathway and gene set visualizations were generated with DittoSeq v1.4.4 and pheatmap v1.0.12.

### Module score calculation

Module scores were calculated using the AddModuleScore function in Seurat. Group differences were assessed using the Wilcoxon rank-sum test, and *p*-values were adjusted by the Benjamini–Hochberg method. Gene sets for chemokine (*CCL2*, *CCL3*, *CCL4*, *CCL7*, *CCL20*, *CCL23*, *CXCL8*), extracellular matrix (ECM) remodeling (*S100A4*, *MMP9*, *ITGA4*, *ITGB2*, *LGALS3*, *COL1A1*, *COL1A2*), TNF-superfamily (*TNF*, *NFKB1*, *RELA*, *NFKBIA*, *TNFAIP3*, *TNFSF13*), and exhaustion (*PDCD1*, *LAG3*, *HAVCR2*, *TIGIT*, *TOX*, *ENTPD1*) pathways were used.

### Trajectory analysis

Pseudotime analysis was performed using Slingshot v2.12.0 based on UMAP embeddings and Seurat-derived clusters, as previously described (35). RNA velocity analysis was performed using Velocyto (v0.17.16) and scVelo (v0.3.3) on loom files generated from CellRanger BAMs aligned to the CanFam4. Spliced/unspliced transcripts were quantified, normalized in Scanpy, and velocities were estimated with the dynamical model on the Seurat-derived UMAP embedding. Naïve T and B cells were specified as the trajectory root. Multiple lineages were inferred, and gene expression dynamics were analyzed along lineage-specific pseudotime axes. The statistical significance of the gene expression trends along pseudotime was assessed using generalized additive models (GAMs), and FDR-adjusted *p*-values < 0.05 were considered significant.

### Cell cycle analysis

Cell cycle phase was assigned using Seurat’s CellCycleScoring function with the cc.genes.updated.2019 gene set, following established protocols (31, 32).

### Cell-to-cell interaction analysis

Intercellular communication was inferred from scRNA-seq data using the CellChat R package v2.1.2 (41). Group-specific CellChat objects were merged into a single master object for comparative analysis. Overall, signaling networks were visualized with the netVisual_heatmap function. To identify and display significant ligand–receptor pairs, we applied the subsetCommunication and netVisual *_*bubble functions using thresholds of ligand log_2_-fold change > 0.2, as well as a receptor log_2_-fold change > 0.1, and *p* < 0.01.

### Statistical methods and reproducibility

Statistical tests were performed primarily within Seurat v5.1.0. For single-cell differential expression analyses, only the non-parametric Wilcoxon rank-sum test was used via FindAllMarkers and FindMarkers. One-way ANOVA and two-tailed unpaired Student’s t-tests (with Welch’s correction when appropriate) were used exclusively to compare log-normalized summary metrics, such as quality control metrics, module scores, and cell proportions, after confirming approximate normality. To evaluate the correlation between two gene signature enrichments, both Pearson’s correlation and simple linear regression analyses were performed using R. Pearson’s correlation coefficient (r) and its statistical significance were computed using the cor.test() function. A simple linear regression analysis was performed using the lm() function. Results were considered statistically significant at *p* < 0.05.

### Data and code availability

The raw and processed scRNA-seq data generated in this study have been deposited in the NCBI Gene Expression Omnibus (GEO) under accession number GSE301630. All custom scripts and pipelines used in this study are publicly available in a GitHub repository at https://github.com/raiora881030-svg/canFam4-canine-PBMC-scRNAseq-scripts. This repository contains the complete R script used for preprocessing, analysis, and visualization of the scRNA-seq data.

## Results

### Clinical and laboratory evaluations of the health status of the dogs

Six healthy dogs (median age, 9.3 years), including Maltese (n = 2), Poodle (n = 1), and mixed-breed dogs (n = 3), were enrolled following normal physical, hematologic, and biochemical examinations (**Supplementary Data 1, 2**). All dogs tested negative for infectious diseases and remained clinically healthy for at least six months under veterinary follow-up.

### The study workflow and the quality control of the data

**Figure 1:**
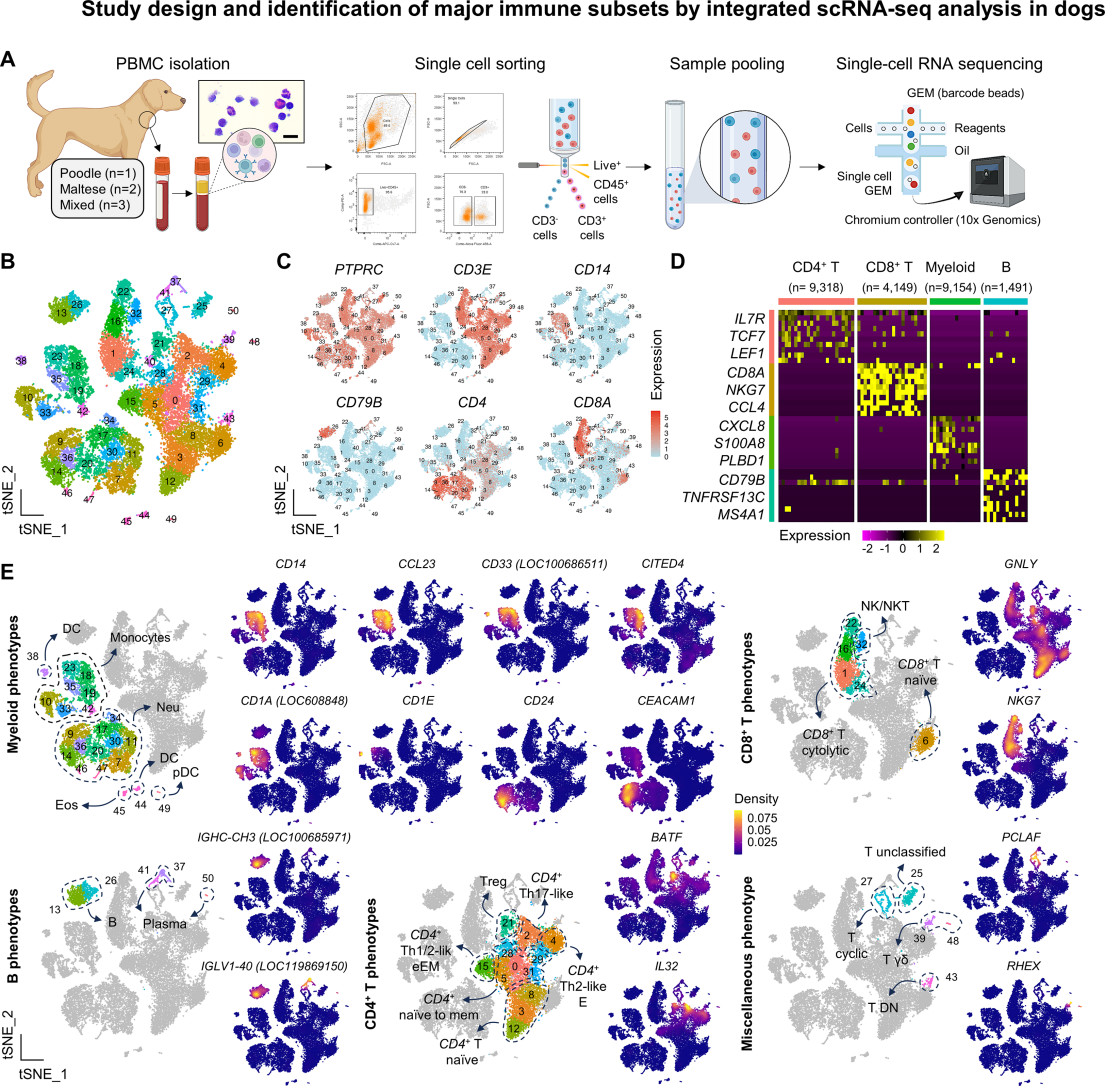
Study design and identification of major immune cell clusters by integrated scRNA-seq analysis in dogs. **(A)** Schematic overview of the study. Live, single CD3^+^ and CD3^-^ peripheral immune cells were isolated from the jugular vein of 6 clinically healthy dogs, flow-sorted, pooled, and subject to single-cell library preparation and downstream bioinformatic analysis. A representative photomicrograph of a hematoxylin and eosin-stained PBMC smear is shown at 400× magnification (scale bar = 20 μm). Scientific illustrations were created using BioRender under an academic license. Please note that the cellular proportions shown in the figures reflect enrichment and do not physiologically represent the actual proportions of immune cells in canine peripheral blood. **(B)** A total of 51 transcriptionally distinct clusters were identified in the integrated Seurat object and visualized on a tSNE plot. **(C)** Canonical lineage markers used to classify major immune cell types are shown on the tSNE feature plot. **(D)** Representative genes among the top 10 DEGs for each major lineage are visualized on a heatmap. The number of cells analyzed per lineage is indicated below. **(E)** The t-SNE density plots of the representative canonical marker genes used to identify functionally distinct immune subsets corresponding to the clusters shown in **Figure 1B**. All marker genes were annotated only in the canFam4 genome reference.

An overview of the study design is provided in **Figure 1A**. A summary of scRNA-seq library construction and multiplexing statistics is provided in **Supplementary Data 3**. Single-cell transcriptomes from six dogs yielded 30,040 high-quality immune cells after standard preprocessing (**Supplementary Figure 1A–E**). Dimensional reduction confirmed comparable transcriptomic structures across breed, age, and sex, ensuring the reliability of pooled analysis. Approximately 15.6% of potential doublets were identified and removed to ensure data integrity for downstream analyses (**Supplementary Figures 1F, G**).

### Identification of major immune subsets in circulating leukocytes of healthy dogs

After singlet selection, we identified 25,355 high-quality cells across 51 clusters (**Figure 1B**). All immune subsets were assigned to each biological replicate (**Supplementary Figure 1H**). Clustering revealed distinct T, B, and myeloid lineages (**Figure 1C**), with representative marker genes defining each major subset (**Figure 1D**). Representative clustering genes used to define functionally distinct immune subsets were present (**Supplementary Figure 2** and **Supplementary Data 4**). The unbiased cell type recognition corroborated our cell type annotation through an enrichment analysis using signatures that define canine and human immune subsets (**Supplementary Figures 3A, B**). Overall, cell type and subset identification in this study supported previous scRNA-seq results applied to circulating leukocytes of healthy dogs (8–10, 23), identifying major functional subsets, such as regulatory T cells (Tregs) (*IL2RA*^+^*FOXP3*^+^), gamma delta (γδ) T cells (*RHEX*^+^*SCART1*^+^), plasmacytoid dendritic cells (pDC) (*IL3RA*^+^*TCF4*^+^), plasma cells (*MZB1*^+^*JCHAIN*^+^), and cycling T cells (cluster 27), characterized by high *MKI67* and *PCLAF* expression and a high proportion of cells in S and G2/M phase (**Supplementary Figure 4**). It is worth noting that adopting the updated canFam4 enables the discovery of features not reported in previous scRNA-seq studies in dogs, including *CD14*, *CCL23*, *CEACAM1*, and *NKG7*, which improved annotation of monocyte, dendritic, and lymphocytic populations (**Figure 1E**).

We then conducted a more comprehensive analysis to investigate reference-specific highly variable features across genome builds. Using the same preprocessing, quality control, and integration pipelines, we applied canFam3.1 to generate a new master Seurat object. Interestingly, we identified 1,490 highly variable features specific to canFam4 (**Supplementary Data 5**), which were expressed across diverse immune subsets (**Supplementary Figure 5A**). In addition to revealing novel genes, canFam4 improved scRNA-seq quality control metrics (**Supplementary Figure 5B**). When applied to the Seurat object, canFam4 significantly increased the number of assigned cells and decreased the proportion of non-human homologous genes. The number of RNA features and the proportion of mitochondrial genes remained consistent across references. In summary, we generated scRNA-seq libraries representing functionally distinct immune subsets in healthy dogs and improved transcriptional resolution and single-cell assignment accuracy by utilizing the canFam4 reference.

### Identification and characterization of myeloid subsets

**Figure 2:**
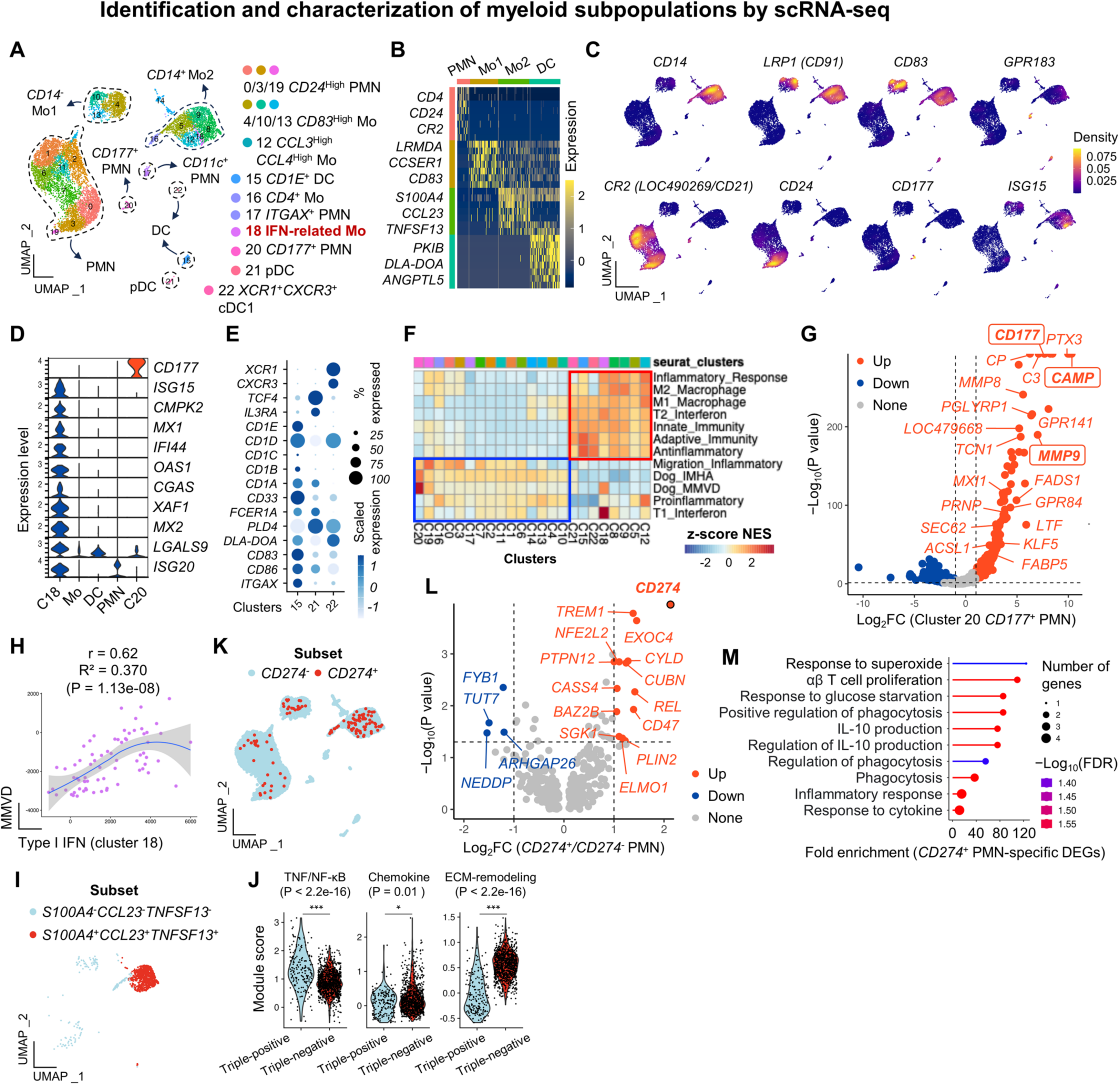
Identification and characterization of myeloid subpopulations by scRNA-seq. **(A)** UMAP visualization of 23 myeloid subsets classified into classical (*CD14*^+^) and non-classical (*CD14*^-^) monocytes, granulocytes (PMNs), and dendritic cells (DCs). Representative subsets are labeled. **(B)** Heatmap of the representative genes among the top 7 DEGs defining each cell type. **(C)** Density plots of the selected marker genes distinguishing functionally distinct myeloid subsets. **(D)** Violin plots of the clusters 18 and 20, characterized by the upregulation of the IFN-related genes and *CD177*, respectively. **(E)** Dot plot of the canonical markers for functional dendritic cell subsets. **(F)** GSEA. Immune-related pathways are enriched in DC/classical monocyte clusters (red box; e.g., M1/M2 macrophage, IFN signatures) and in PMN/non-classical monocyte clusters (blue box; e.g., migration, Dog_IMHA, Dog_MMVD signatures). **(G)** Volcano plot of the DEGs in *CD177*^+^ PMNs compared to other PMN subsets. Genes associated with Dog_MMVD are highlighted. **(H)** Scatter plot of the correlation between type I IFN and MMVD gene signature scores in cluster 18. **(I)** Feature plot of the distribution of *S100A4*^+^*CCL23*^+^*TNFSF13*^+^ monocytes and their negative counterparts. **(J)** Module score analysis of the TNF/NF-κB, chemokine, and ECM-remodeling gene programs between triple-positive (*S100A4*^+^*CCL23*^+^*TNFSF13*^+^) and triple-negative subsets. **(K)** Feature plot of the distribution of *CD274*^+^ PMNs and monocytes. **(L)** Volcano plot of the representative DEGs in *CD274*^+^ PMNs compared to *CD274*^-^ PMNs. *CD274* is highlighted with a log_2_FC of 16.4 and *p* = 0. **(M)** GO analysis of *CD274*^+^ PMN-specific DEGs reveals enrichment in biological processes associated with T cell proliferation, glucose starvation, phagocytosis, and IL-10 production. The *p*-values were derived using one-way analysis of variance implemented in the ggpubr R package, along with Wilcoxon rank-sum tests for pairwise comparisons. * *p* < 0.05, ** *p* < 0.01, and *** *p* < 0.001. Abbreviations: PMNs, polymorphonuclear cells; FC, fold change; DEGs, differentially expressed genes; C, cluster; Mo, monocytes; DCs, dendritic cells; pDCs, plasmacytoid DCs; GO, Gene Ontology; GSEA, gene set enrichment analysis.

Contrary to humans, granulocytes, polymorphonuclear cells (PMNs), or myeloid-derived suppressor cells (MDSCs) are collected during gradient-based isolation of canine peripheral blood (8, 10, 42). Canine myeloid population is highly heterogeneous; however, our understanding of this population at the single-cell level remains incomplete (8–10, 23). Therefore, we first focused on identifying and characterizing functionally distinct myeloid subsets. Sub-clustering of *DPYD*^+^ myeloid cells revealed 23 clusters in dogs (**Figure 2A**). Representative marker genes used for functional classification are summarized in **Supplementary Figure 6** and **Supplementary Data 6**.

Canine myeloid populations comprised granulocytes, monocytes, and dendritic cell (DC) subsets, each exhibiting distinct transcriptional profiles defined by canonical markers such as *CD4*, *CD24*, *CR2* (*CD21/LOC490269*), *S100A4*, *CD83*, *CCL23*, *TNFSF13*, and *DLA*-*DOA* (**Figure 2B**). Among monocytes, we identified *S100A4*^+^*CCL23*^+^*TNFSF13*^+^*CD14*^+^ clusters representing classical monocytes (clusters 5, 8, 9, 12, 16, and 18) (**Figure 2C**), which were not previously reported in canine scRNA-seq datasets (8–10, 23). In contrast, clusters 4, 10, and 13 expressing *CD83* and *CD91* (*LRP1*) likely corresponded to non-classical or transitional subsets (12, 43). The *CD16* or *FCGR3A* transcript was found to be unannotated in canFam4. A cluster 8 characterized by high expression of interferon (IFN)-stimulated genes (ISG), such as *ISG15*, *MX1*, *MX2*, *IFI44*, *OAS1*, and *XAF1*, represented IFN-stimulated monocytes (**Figure 2D**) (31). A majority of the IFN-related monocytes showed *LGALS9* expression (**Supplementary Figure 6B**). DC subsets (clusters 15, 21, and 22) expressed *GPR183*, *DLA*-*DOA*, *CD86*, and *PLD4*, with all CD1 family genes (*CD1A*-*E*) differentially expressed across DC subtypes (**Figures 2C–E**). Similar to humans, dogs exhibited *XCR1*^+^ terminally differentiated conventional type 1 DCs (cDC1; cluster 22), *TCF4*^+^*IL3RA*^+^ pDC (cluster 21), and *ITGAX*^+^*FCER1A*^+^ cDC2 (cluster 15) subsets (44). The *XCR1*^+^ DC was characterized by significant *MIF* upregulation (**Supplementary Figure 6C**). PMNs were marked by *CD21* (*CR2*/*LOC490269*) and *CD4*, while *CD177*, *ITGAX*, *CEACAM1*, and *CD24* delineated transcriptionally distinct neutrophil clusters (**Figures 2C, E**). Meanwhile, cluster 14 co-expressed both *CD14* and *CD3E*, meaning that this cluster was likely a doublet.

Next, we functionally characterized how these distinct myeloid subsets contribute to immune homeostasis in healthy dogs (**Figure 2F**). First, *CD14*^+^ monocytes and DC subsets were preferentially enriched in inflammatory gene signatures, such as M1/M2 macrophage markers, innate/adaptive immunity, and IFN signaling pathways (**Figure 2F**, red box). In contrast, neutrophil subsets were preferentially enriched for gene sets related to leukocyte migration involved in the inflammatory response (**Figure 2F**, blue box). Second, we found that certain myeloid subsets may be associated with immune dysregulation of canine diseases. For instance, *CD177*^+^ neutrophils showed strong enrichment in gene signatures associated with immune-mediated hemolytic anemia (IMHA) (36) and myxomatous mitral valvular disease (MMVD) (45). There was also a significant positive correlation between IMHA and MMVD signatures in the *CD177*^+^ neutrophils (**Supplementary Figure 6E**). Genes such as *CD177*, *CAMP*, and *MMP9* were significantly upregulated in these neutrophils (**Figure 2G**). IFN-related monocytes also exhibited MMVD signature enrichment, with notable upregulation of *ISG15* and *MX1* (**Supplementary Figure 6F**). Additionally, a moderately strong, significant positive correlation was observed between type I IFN and MMVD gene signatures (**Figure 2H**). Finally, we further characterized previously unreported *CD14*^+^ monocytes defined by *S100A4*^+^*CCL23*^+^*TNFSF13*^+^expression. Among *CD14*^+^ monocytes, the triple-positive and -negative subsets were separated and subjected to module score analyses for chemokine, ECM remodeling, and TNF superfamily programs (**Figure 2I**). Interestingly, despite high expression of *TNFSF13*, the triple-positive *CD14*^+^ monocytes lacked a global TNF/NF-κB activation signature, while exhibiting upregulated chemokine and ECM-remodeling gene programs (**Figure 2J**).

Anti-PD-L1 antibodies have shown anti-tumor activity in dogs with naturally occurring cancers (46, 47); however, their immunological mechanisms of action in dogs remain insufficiently characterized. We investigated the contribution of *CD274*, which encodes PD-L1 protein, in myeloid-mediated immune regulation. At steady-state, *CD274* was expressed in neutrophils and monocytes but not in DCs (**Figure 2K**, **Supplementary Figure 6G**). We separated myeloid cells based on *CD274* expression and performed differential gene expression analysis. *CD274*^+^ neutrophils exhibited 18 differentially expressed genes compared to *CD274*^-^ neutrophils (**Figure 2L**, **Supplementary Data 7**). GO analysis of these DEGs revealed enrichment in pathways such as αβ T cell proliferation, IL-10 production, phagocytosis, and the inflammatory response, supporting the functional phenotype of PMN-MDSCs (48) (**Figure 2M**, **Supplementary Data 8**). Notably, *CD47*, a part of the IL-10 signature, was significantly upregulated in *CD274*^+^ neutrophils. Similarly, 172 DEGs defined *CD274*^+^ monocytes (**Supplementary Figure 6G**), which were enriched in PD-L1 checkpoint, toll-like receptor, NF-κB, and C-type lectin receptor pathways (**Supplementary Figure 6I** and **Supplementary Datas 7**, **8**). Taken together, we identified functionally distinct myeloid subsets, demonstrating their potential clinical relevance in regulating immune homeostasis in dogs.

### Identification and characterization of *CD4*^+^ T subsets

**Figure 3:**
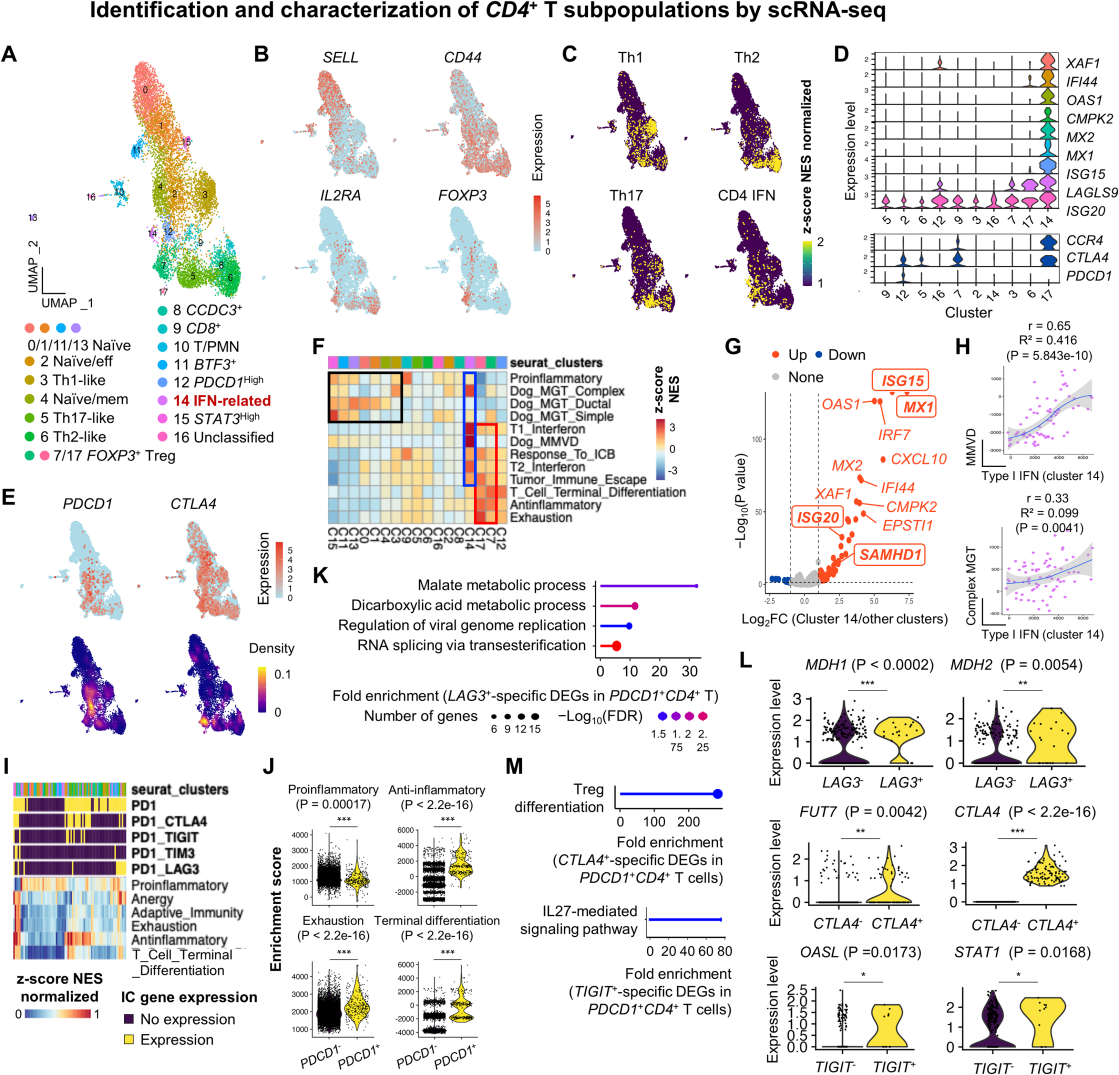
Identification and characterization of *CD4*^+^ T subpopulations by scRNA-seq. **(A)** UMAP plot of the 18 distinct *CD4*^+^ T cell subsets. **(B)** Cell type recognition using the escape R package. Feature plots show enrichment of canine Th1, Th2, and Th17 gene signatures along with *FOXP3* expression. **(C)** Expression patterns of IC genes are shown on the feature plot. **(D)** Violin plots of the distinct gene expression in clusters 14 (IFN-related) and 12 (*PDCD1*^+^*CTLA4*^+^). **(E)** Feature and density plots of the expression patterns of *PDCD1* and *CTLA4* in *CD4*^+^ T cells. **(F)** GSEA of the preferential pathway enrichment in effector *CD4*^+^ T phenotypes (red boxed). IFN-related cluster 14 is strongly enriched for IFN signaling and Dog_MMVD signatures. Treg and *PDCD1*^high^ clusters are enriched for terminal differentiation pathways. **(G)** Volcano plot of the DEGs in IFN-related *CD4*^+^ T cells compared to other clusters. Dog_MMVD-associated genes are highlighted in bold and boxed. **(H)** Feature scatter plots of the positive correlation between two gene signatures in the IFN-related *CD4*^+^ T cells. **(I)** GSEA reveals that *CTLA4*^+^ or *LAG3*^+^*PDCD1*^+^*CD4*^+^ T cells are enriched for anti-inflammatory, anergy, and exhaustion signatures. **(J)** Violin plots of the enrichment pattern of immune-associated gene sets in *CD4*^+^ T cells with or without *PDCD1*. **(K)** GO analysis of *LAG3*-associated DEGs in *PDCD1*^+^*CD4*^+^ T cells reveals enrichment in malate and dicarboxylic acid metabolism, and viral genome replication. **(L)** Violin plots demonstrates upregulation of *LAG3*, *CTLA4*, and *TIGIT*-specific key genes involved in malate metabolism, Treg differentiation, and IL-27-mediated signaling pathways in *PDCD1*^+^*CD4*^+^ T cells, respectively. **(M)** GO analysis of *CTLA4*- and *TIGIT*-specific DEGs in *PDCD1*^+^*CD4*^+^ T cells reveals enrichment in Treg differentiation and IL-27-mediated signaling pathways, respectively. Statistical significance was determined by comparing two groups of interest using the non-parametric Wilcoxon rank-sum test. * *p* < 0.05, ** *p* < 0.01, and *** *p* < 0.001. FC, fold change; DEGs, differentially expressed genes; C, cluster; Tregs, regulatory T cells; GSEA, gene set enrichment analysis; GO, Gene Ontology.

T helper subsets play major roles in canine health and disease (49). We next identified and characterized *CD4*^+^ T cell subsets in healthy dogs (**Figure 3A**). Cell identities were assigned using canonical markers and canine *CD4*^+^ T cell references (8, 22, 24), with representative marker genes summarized in **Supplementary Figure 7A** and **Supplementary Data 9**. Naïve (*SELL*^+^*CD44*^-^) (clusters 0, 1, 11, 13, and 15) and effector/memory (*SELL*^-^*CD44*^+^) (clusters 2, 3, 4, 5, 6, 7, 8, 12, 14, and 17) populations were clearly separated (**Figure 3B**). Functionally distinct Th1 (cluster 3), Th2 (cluster 6), and Th17 (cluster 5) subsets were identified, exhibiting partial transcriptional overlap (**Figure 3C**). A transcriptionally unique IFN-related subset (cluster 14) displayed strong enrichment of ISGs, including *XAF1, OAS1, MX1*, and *ISG15* (**Figures 3C, D**). *PDCD1*^high^ cluster 12 represented a putative exhausted phenotype (**Figure 3E**). Tregs (clusters 7 and 17) expressed canonical *FOXP3*, *IL2RA*, *CCR4*, and *CTLA4* alongside *BATF* and *IL32* (**Supplementary Figures 7A, B**). *CTLA4* was broadly expressed across multiple *CD4*^+^ subsets (**Figure 3E**). A small *CD8A*^+^ cluster 9 indicated double-positive T cells, while clusters 8, 11, and 15 remained transcriptionally unclassified but expressed *CCDC3, LOC119876465*, and *STAT3*, respectively. Cluster 10, co-expressing *CSF3R, DPYD*, and *S100A8*, was likely a doublet population (**Supplementary Figure 7B**).

We next explored the functional and clinical relevance of these subsets. First, similar to myeloid cells, IFN-related *CD4*^+^ T cells exhibited marked enrichment of canine MMVD gene signature (blue boxed in **Figure 3F**), characterized by the upregulation of *MX1* and *ISG15* (**Figure 3G**) and a positive correlation between type I IFN and MMVD signatures (**Figure 3H**, upper panel). A similar pattern was observed in the IFN-related subset regarding mammary complex carcinoma signature, showing upregulation of *ISG20* and *SAMHD1*, along with a corresponding gene set correlation (**Figures 3F–H**, lower panel). Second, Treg clusters were highly enriched in anti-inflammatory signatures, consistent with their immunosuppressive roles in healthy dogs (50) (red boxed in **Figure 3F**). Interestingly, upon subclustering of Tregs based on *CCR4* expression (**Supplementary Figure 7C**), *CCR4*^+^ Tregs were more enriched with gene sets associated with tumor immune evasion and response to immune checkpoint (IC) blockade (**Supplementary Figure 7D**), suggesting that CCR4^+^ Tregs possess a regulatory program that could become clinically relevant under pathological conditions such as cancer (51, 52). DEGs defining *CCR4*^+^ Tregs compared to *CCR4*^-^ ones were obtained and subjected to GO analysis (**Supplementary Data 10**), which revealed significant enrichment with the gene set associated with tRNA wobble base modification (**Supplementary Data 11**). Among genes listed in the signature, *CCR4*^+^ Tregs showed a significant upregulation in *NSUN3*, *ELP2*, *GTPBP3*, *ELP3*, and *CTU1* but *ADAT2* downregulation. Third, *PDCD1*^high^*CD4*^+^ T cells showed modest enrichment of gene signatures associated with terminal T cell differentiation and exhaustion (**Figure 3F**), suggesting that healthy dogs have *CD4*^+^ T cells undergoing transition toward an exhausted phenotype. Finally, *CD4*^+^ T subsets enriched for mammary gland tumor-associated gene signatures included several naïve, Th1-like, and *STAT3*^high^ proinflammatory populations (black boxed in **Figure 3F**).

In healthy dogs, PD1 blockade has been shown to reverse CD4^+^ T cell suppression induced by tumor-derived PD-L1 (53), but the underlying molecular mechanisms are not well understood. We therefore analyzed the transcriptional profiles of *CD4*^+^ T cells expressing IC genes. *CD4*^+^ T cells with or without *PDCD1* expression exhibited different enrichment patterns for gene signatures associated with immune activation and exhaustion (**Figure 3I**). Notably, compared to *PDCD1*^-^*CD4*^+^ T cells, *PDCD1*^+^*CD4*^+^ T cells showed significant enrichment of exhaustion-related gene signatures, suggesting an inhibitory effect of *PDCD1* on *CD4*^+^ T cell immunity (**Figure 3J**). Similarly, *PDCD1*^+^*CD4*^+^ T cells showed downregulation of T cell receptor (TCR)-related genes (*ID3*, *CCR7*, *FOXP1*, and *TRAT1*) but also showed upregulation of genes such as *BATF*, *ISG20*, *IFI30*, *ITGB1*, and *IDH2* (**Supplementary Data 12**). Recently, a dog with anti-PD-L1-resistant melanoma exhibited a complete remission of an oral neoplastic lesion following anti-CTLA4 therapy (54). To further elucidate the mechanism of action, we performed differential gene expression and gene set enrichment analyses, which revealed that *CTLA4*-specific genes, such as *FOXP3*, *TIGIT*, *HAVCR2*, *IL10*, *ADORA2A*, and *ADA* (**Supplementary Figure 7E** and **Supplementary Data 13**), were significantly upregulated and enriched in pathways related to negative regulation of the immune system (**Supplementary Figure 7F**). We next investigated the biological implications of additional IC genes expressed in *PDCD1*^+^*CD4*^+^ T cells. Among this population, distinct subgroups co-expressed additional IC genes, including *CTLA4* (n = 95), *LAG3* (n = 20), *TIGIT* (n = 11), and *HAVCR2* (n = 4). Notably, *PDCD1*^+^*CD4*^+^ T cells co-expressing *TIGIT*, *HAVCR2*, or *LAG3* showed a tendency toward enrichment of an exhaustion signature (**Supplementary Figure 7G**). DEGs associated with each IC gene are provided in **Supplementary Data 14**. Given the robustness of differential expression analysis in scRNA-seq datasets (55), GO enrichment analysis of *LAG3*-, *CTLA4*-, and *TIGIT*-specific DEGs was performed. Interestingly, *LAG3*-specific genes were significantly enriched in biological processes related to malate and dicarboxylic acid metabolism, viral genome replication, and RNA splicing (**Figure 3K**). Within the metabolic gene set, *MDH1* and *MDH2* were significantly upregulated in *LAG3*^+^*PDCD1*^+^*CD4*^+^ T cells compared to *LAG3*^-^ counterparts (**Figure 3L**). Similarly, *CTLA4*- and *TIGIT*-specific genes were enriched in pathways associated with Treg differentiation and IL-27 signaling, respectively (**Figure 3M**). In these gene sets, *FUT7*, *OASL*, and *STAT1* were notably upregulated in *IC*^+^*PDCD1*^+^*CD4*^+^ T cells (**Figure 3L**). Collectively, our results show the functional diversity and IC heterogeneity of *CD4*^+^ T cell subsets in dogs, highlighting their potential clinical relevance in immune homeostasis.

### Identification and characterization of *CD8*^+^ and other T subsets

**Figure 4:**
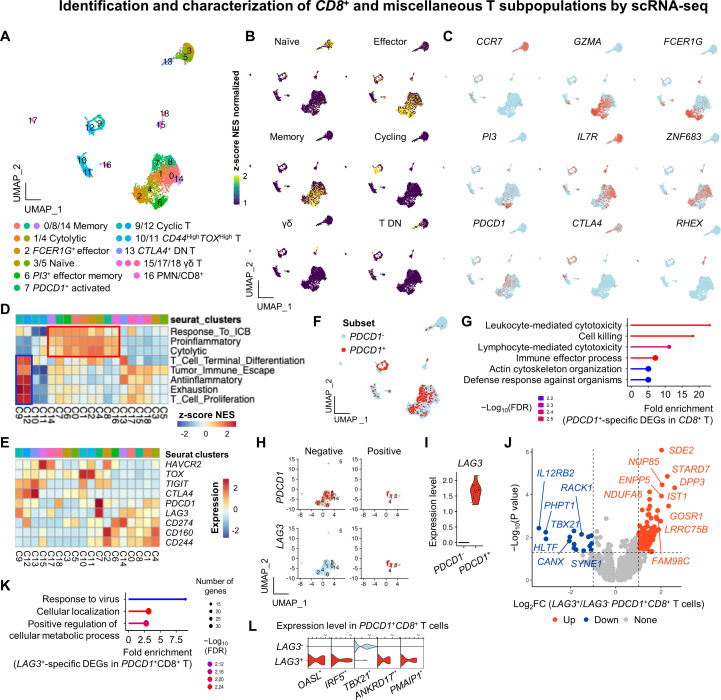
Identification and characterization of *CD8*^+^ and miscellaneous T subpopulations by scRNA-seq. **(A)** UMAP plot of the ten *CD8*^+^ T, three γδ T, two cycling T, and three unclassified T cell subsets. **(B)** Feature plots of the enrichment of canine gene signatures associated with naïve, effector, memory, cycling, γδ, and T DN T cells. **(C)** Feature plot of representative marker gene expression. **(D)** GSEA of the enrichment of immune-associated pathways across *CD8*^+^ T subsets. Effector phenotypes (red box) and cycling T subsets (blue box) are enriched in proliferation and exhaustion-related signatures. γδ T cells exhibit mild enrichment in naïve, exhausted, and proliferative signatures. Clusters 2, 4, and 7 show enrichment in cytolytic, proinflammatory, memory, and terminal differentiation pathways. **(E)** Heatmap of the genes associated with T cell terminal differentiation. *PDCD1* and *LAG3* are highly expressed in cluster 7. **(F)** Feature plot of the *PDCD1*^+^ T cell distribution. **(G)** GO analysis of *PDCD1*-specific DEGs reveals enrichment of effector function and cytotoxicity-related biological processes. Feature **(H)** and violin **(I)** plots of the *LAG3*^+^*PDCD1*^+^*CD8*^+^ T cells. **(J)** Volcano plot of *LAG3*-specific DEGs in *PDCD1*^+^*CD8*^+^ T cells. **(K)** GO analysis of the *LAG3*-specific DEGs in *PDCD1*^+^*CD8*^+^ T cells shows enrichment in antiviral response processes. **(L)** Violin plots of the representative genes in the antiviral response pathway. All genes except *TBX21* are significantly upregulated in *PDCD1*^+^*CD8*^+^ T cells. Statistical significance was assessed using the Wilcoxon rank-sum test. * *p* < 0.05; ** *p* < 0.01. PMNs, polymorphonuclear cells; FC, fold change; DEGs, differentially expressed genes; C, cluster; T DN, double-negative T; GO, Gene Ontology.

We next performed subclustering of non-*CD4*^+^ T cells, identifying ten transcriptionally distinct *CD8*^+^ T clusters (0, 1, 2, 3, 4, 5, 6, 7, 8, and 14), three γδ T clusters (15, 17, 18), two cycling T clusters (9, 12), and one *CD4*^-^*CD8A*^-^ double-negative (DN) cluster (13) (**Figure 4A**). Representative canonical and cluster markers are summarized in **Supplementary Figure 8A** and **Supplementary Data 15**. Based on established canine *CD8*^+^ T cell signatures (8, 22, 24), naïve *CD8*^+^ T cells (clusters 3 and 5) expressed *CCR7, SELL, LEF1*, and *TCF7*. In contrast, effector *CD8*^+^ T cells (clusters 0, 1, 2, 4, 6, 7, 8, 14) displayed high expression of cytotoxic genes, including *GZMB, GZMH* (*LOC490629*), *GZMK*, and *PRF1* (**Figures 4B, C**, **Supplementary Figures 8A, B**). Innate-like subsets (clusters 2 and 6) co-expressed *FCER1G* and *PI3* (9), with cluster 6 further enriched in *CXCR6* and effector-memory markers (*PRDM1, ZNF683, STAT3*), consistent with a mucosal-associated invariant T cell-like phenotype. *PDCD1*^+^*LAG3*^+^ effector T cells (cluster 7) and cycling/*FOXP3*^+^*CTLA4*^+^ subsets (clusters 9, 12, 13) were also identified, reflecting activation or regulatory states (**Figure 4D**). Interestingly, the *CTLA4* expression was largely confined to cycling T (51.6% and 59.7% in clusters 9 and 12, respectively) and DN T cells (54.4% in cluster 13). γδ T cells expressed *RHEX* and *SCART1 (LOC491694)*, while unclassified clusters 10 and 11 displayed *CD44, TOX*, and *LOC102155278*. Cluster 16, co-expressing *CD8A, CSF3R*, and *DPYD*, was likely a doublet.

Functional characterization using GSEA revealed several distinct patterns. First, effector *CD8*^+^ T cells were mainly enriched in proinflammatory, cytotoxic, and response to IC blockade gene sets (red boxed in **Figure 4D**), indicating functional competence, such as anti-tumor immunity in dogs (56). Second, T cell activation-associated gene signatures were markedly enriched in cycling T cells (blue boxed in **Figure 4D**), suggesting that cycling T cells may represent a state of steady-state antigenic stimulation (57). Third, although previous studies reported exhausted *CD8*^+^ T subsets in healthy dogs (9), our data showed that *PDCD1*^+^*CD8*^+^ T cells, despite expressing co-inhibitory receptors and being enriched for the T cell terminal differentiation signature (**Figure 4E**), did not exhibit remarkable enrichment of an exhaustion signature (**Supplementary Figure 8C**). Fourth, γδ T subsets also showed enrichment in T cell activation along with tumor immune escape signatures, suggesting a potential role in tumor immune surveillance. Lastly, although positioned adjacent to naïve T subsets on the dimensional plot, the DN T subset was enriched for anti-inflammatory and T cell terminal differentiation gene signatures, along with remarkable *CTLA4* and *TIGIT* expression. Additional pseudotime analysis revealed that the DN T subset was localized near the root of the trajectory and was not associated with any terminal lineage (**Supplementary Figures 8D**). Gene expression dynamics along eight terminal lineages revealed that *CTLA4* was transiently upregulated during early pseudotime in multiple lineages, particularly in cell fate 1 and 2 (**Supplementary Figure 8F**), suggesting DN T subset as an initial activation phase before differentiation.

To further explore the immunoregulatory role of *PDCD1* and co-inhibitory receptors in *CD8*^+^ T cells, we performed DEG analysis between *PDCD1*^+^*CD8*^+^ and *PDCD1*^-^*CD8*^+^ T cells (**Figure 4F**). *PDCD1*-specific DEGs (**Supplementary Data 16**) were enriched in immune effector pathways such as cytotoxicity, cell killing, and defense response (**Figure 4G** and **Supplementary Data 17**). Genes, such as *BATF*, *SH2D1A*, *CORO1A*, *PRF1*, and *GZMB* were commonly upregulated, while *IL7R* was downregulated. Meanwhile, similar to *CD4*^+^ T cells, *PDCD1* expression was significantly associated with an inhibitory effect on *CD8*^+^ T cell immunity (**Supplementary Figure 8F**). To further investigate functional implications of IC genes, *PDCD1*^+^*CD8*^+^ T cells were sub-grouped based on the expression of *LAG3* (n = 20) (**Figures 4H, I**), *HAVCR2* (n = 8), *CTLA4* (n = 3), and *TIGIT* (n = 4). Considering robustness of differential expression analysis in scRNA-seq datasets, we focused on *LAG3*-specific DEGs for downstream functional analysis (**Figure 4J**, **Supplementary Data 18**). GO enrichment revealed a significant association with viral response pathways (**Figure 4K**). Among the enriched genes, *PMAIP1*, *ANKRD17*, *IRF5*, and *OASL* were significantly upregulated, whereas *TBX21* was downregulated (**Figure 4L**). Meanwhile, *PDCD1*^+^*CD8*^+^ T cells with *CTLA4* and *LAG3* expression, showed a tendency for the enrichment of an exhaustion signature (**Supplementary Figure 8G**). We next validated our findings using publicly available scRNA-seq datasets from external cohorts of canine osteosarcoma. Using the same procedures for preprocessing, quality control, data integration, and doublet removal as applied to our own datasets, we subclustered *CD8*^+^ T cells and classified them according to *PDCD1* and subsequently *LAG3* expression (**Supplementary Figures 9A, B**). *PDCD1*^+^*CD8*^+^ T cells (n = 36) exhibited higher module scores of cytotoxicity, proliferation, and exhaustion compared with their *PDCD1*^-^ counterparts (n = 4240) (**Supplementary Figure 9C**). Notably, *LAG3*^+^*PDCD1*^+^*CD8*^+^ T cells (n = 24) showed elevated module scores of both exhaustion and response to viral programs, further supporting our findings (**Supplementary Figure 9D**). Taken together, our results reveal transcriptionally distinct *CD8*^+^ T cell subsets in healthy dogs, with functional profiles supporting roles in immune homeostasis.

### Identification and characterization of B subsets

**Figure 5:**
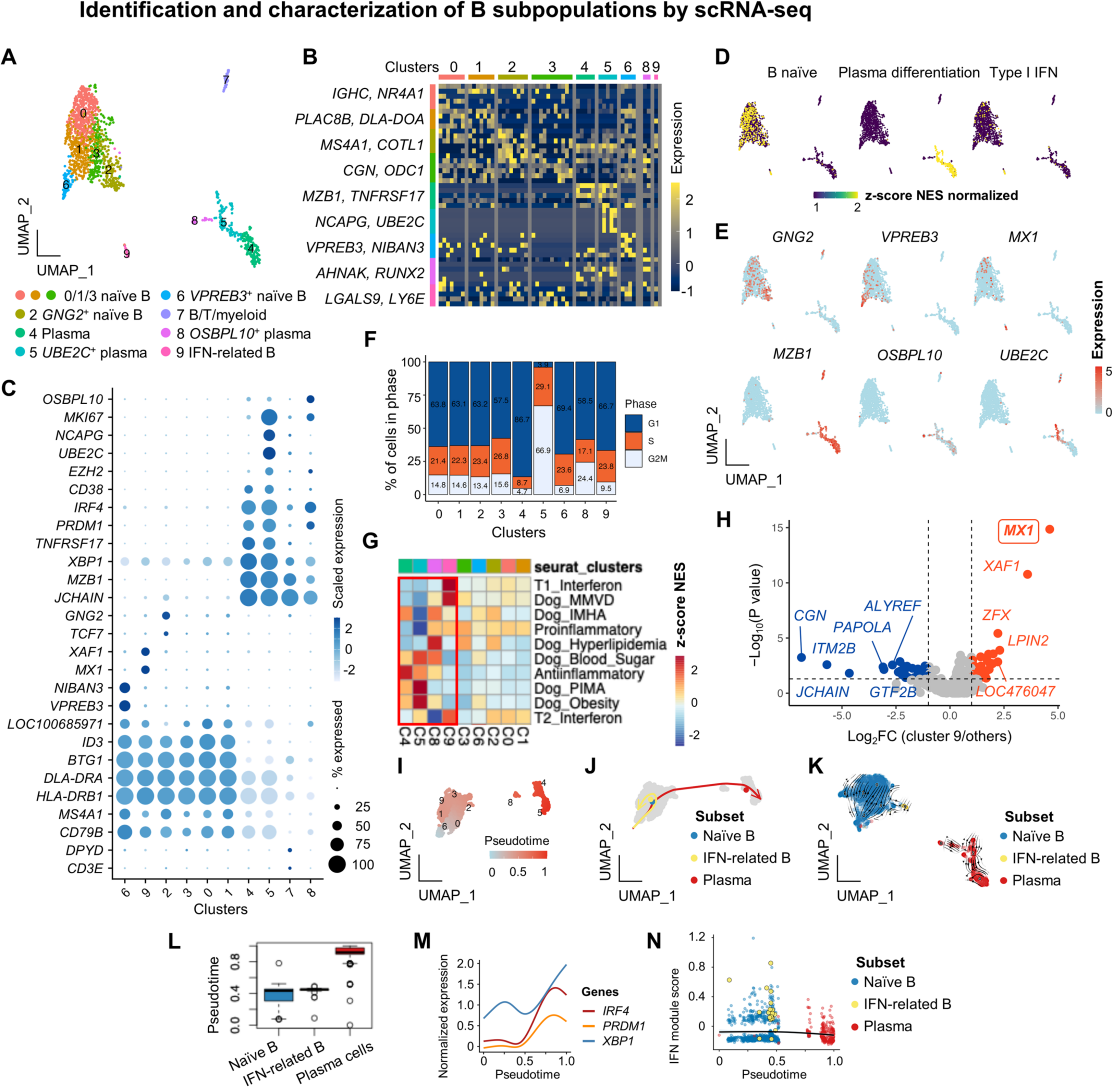
Identification and characterization of B subpopulations by scRNA-seq. **(A)** UMAP plot of the six B cell and three plasma cell subsets. **(B)** Heatmap of the top five DEGs defining each subset. **(C)** Feature plots of the enrichment of naïve and plasma cell gene signatures, along with representative marker expression. **(D)** Dot plot of the canonical markers used to identify functionally distinct B cell subsets. **(E)** Cell cycle phase distribution across B cell subsets. **(F)** GSEA of the enrichment patterns of immune-related pathways across B cell subsets. **(G)** Volcano plot of the DEGs in IFN-related B cells compared to other B cell subsets. *MX1*, listed in the Dog_MMVD signature, is highlighted in bold and boxed. **(I)** Pseudotime ordering of B-cell subsets. **(J)** Trajectory inference showing bifurcated differentiation paths from naïve B cells toward IFN-related or plasma fates. **(K)** RNA velocity analysis showing directional flow from naïve B cells toward IFN-related and plasma subsets. Streamlines indicate the inferred transcriptional dynamics based on spliced and unspliced mRNA ratios. **(L)** Distribution of pseudotime values across B cell subtypes. **(M)** Expression dynamics of plasma-associated regulators (*IRF4*, *PRDM1*, *XBP1*) along the naïve–plasma trajectory. All three genes showed significant non-linear associations with pseudotime by GAM analysis (FDR < 0.001). **(N)** IFN module scores plotted along pseudotime, showing selective activation in IFN-related B cells (GAM, FDR < 0.05).

We performed subclustering of *CD79B*^+^ B cells and *MZB1*^+^ plasma cells, identifying ten transcriptionally distinct clusters (**Figure 5A**). Canonical and cluster-defining genes are presented in **Figures 5B, C**, and representative markers are summarized in **Supplementary Data 16**. Unbiased annotation using canine B cell references (8, 22, 24) classified subsets into naïve (clusters 0, 1, 2, 3, 6, 9) and plasma (clusters 4, 5, 8) cells (**Figure 5D**). Naïve B cells expressed *LOC100685971, CD79B, BTG1, MS4A1*, and MHC class II genes (*HLA-DRB1, DLA-DRA*), whereas clusters 2, 6, and 9 showed additional expression of *GNG2, VPREB3*, and *MX1*, consistent with type I IFN-related B cells (**Figures 5D, E**). Plasma subsets were characterized by *JCHAIN, MZB1, PRDM1*, and *XBP1* but lacked MHC class II expression (*DLA*-*DMB, DLA*-*DOA*). Distinct transcriptional identities were observed in *UBE2C*^+^ and *OSBPL10*^+^ plasma subsets, with the *UBE2C^+^* subset displaying a high G2/M cell-cycle score, suggesting proliferative activity under steady-state conditions (**Figure 5F**). Cluster 7, co-expressing *CD3E* and *DPYD*, was excluded as a potential doublet.

For functional characterization, GSEA revealed significant enrichment in plasma and IFN-related B subsets (**Figure 5G**, red box). Plasma subsets were enriched with gene sets associated with anti-inflammatory responses and canine immune/metabolic conditions, including IMHA, precursor-targeted immune-mediated anemia (PIMA), obesity, blood sugar, and hyperlipidemia. The IFN-related B subset was highly enriched in canine MMVD signatures, with significant upregulation of *MX1* (**Figure 5H**). A weak positive correlation was observed between type I IFN and MMVD signatures (r = 0.309, *p* = 0.1723), but it did not reach statistical significance. Given these biologically divergent enrichment patterns, we next examined whether the plasma and IFN-related B subsets represent discrete lineages or instead reflect transcriptionally connected states within a shared differentiation continuum. To address this, these subsets were subjected to pseudotime ordering. Trajectory analysis revealed a continuous transition from naïve B cells toward either IFN-related or plasma fates (**Figure 5I, J**), which was further supported by RNA velocity analysis (**Figure 5K**). Naïve and plasma subsets were positioned at opposite ends of the trajectory, while IFN-related B cells occupied an intermediate branch, suggesting bifurcated differentiation (**Figure 5L**). Expression of plasma cell-associated regulators (*IRF4, PRDM1, XBP1*) increased significantly and progressively along the naïve-to-plasma lineage (**Figure 5M**), whereas IFN-related B cells diverged early from the naïve state, selectively upregulating ISG signatures (**Figure 5N**). Collectively, these analyses demonstrate that canine B cells follow distinct differentiation trajectories leading either to antibody-secreting plasma cells or IFN-driven effector states, underscoring substantial transcriptional heterogeneity and functional diversification within the peripheral B cell compartment.

### Cell–cell communication identifies key signaling pathways with ligand and receptor genes in regulating normal homeostasis in healthy dogs

**Figure 6:**
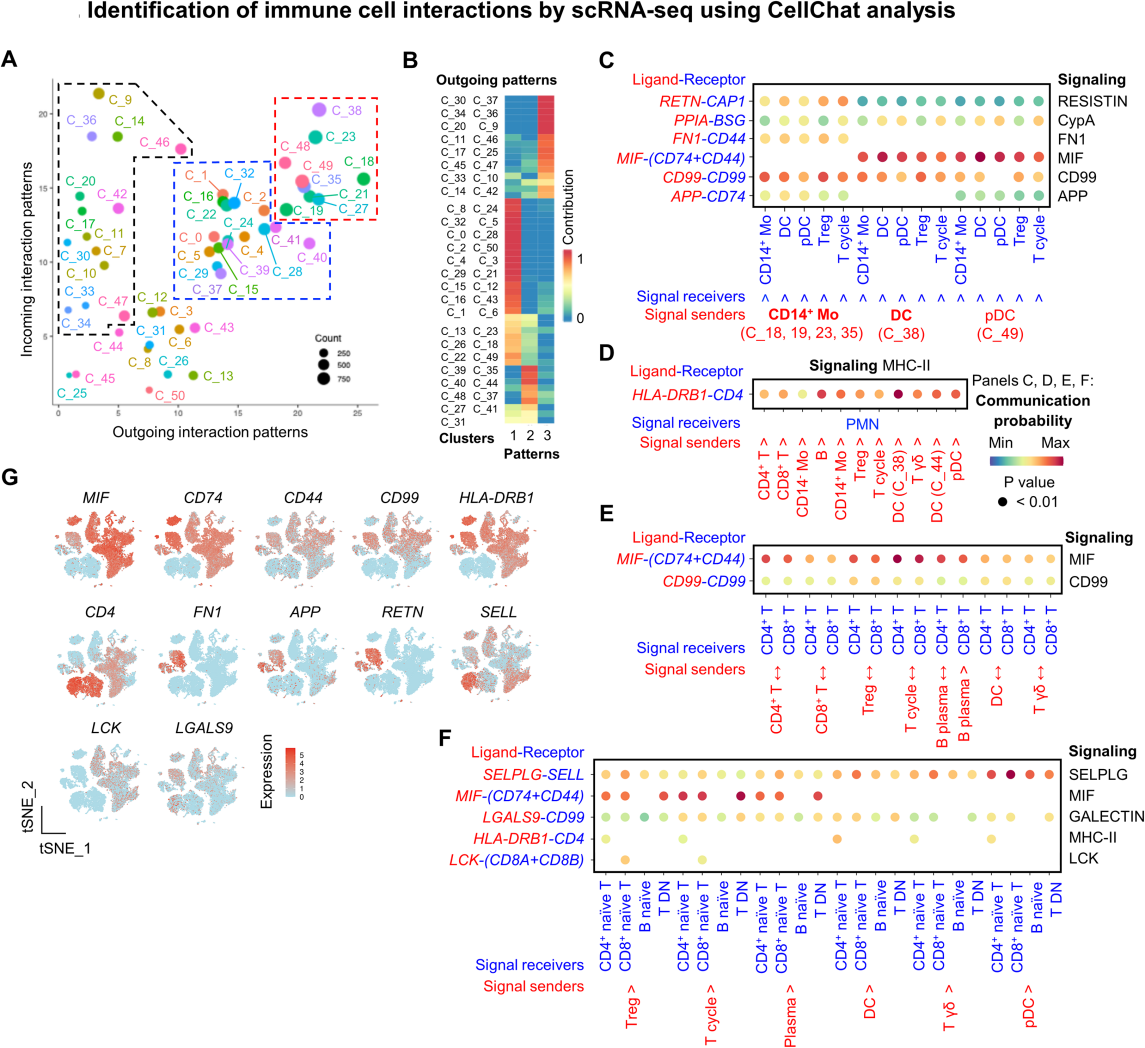
Identification of immune cell interactions by scRNA-seq using CellChat analysis. **(A)** Inferred intercellular communication network highlighting dominant signal senders (red and blue dashed lines) and receivers (black dashed line) visualized on a scatter plot. **(B)** Global outgoing signaling patterns across identified immune cell clusters. **(C–F)** Bubble plots of the significant ligand–receptor interactions involved in representative signaling pathways among indicated immune subsets. **(G)** Feature plots show the expression patterns of representative ligands and receptors involved in MIF, CD99, MHC-II, Selplg, FN1, APP, and galectin signaling pathways.

Coordinated crosstalk among immune subsets is essential for maintaining immune homeostasis (31). To explore this, we investigated the cell-to-cell interactions involved in modulating immune equilibrium in healthy dogs. The overall signaling landscape across immune subsets is shown in **Supplementary Figure 10A**. In healthy dogs, we identified three major patterns of cell-cell interaction (**Figures 6A, B**).

First, autocrine signaling was strongly inferred among myeloid subsets, including DC (cluster 38), pDC (cluster 49), and *CD14*^+^ monocytes (clusters 18, 19, 23, and 35) (**Figure 6A**, red dashed lines, **Supplementary Figure 10A**). These myeloid subsets also transmitted signals to Treg (cluster 21), cycling T cells (cluster 27), and γδ T cells (cluster 48), suggesting a role in modulating T cell activity. The interactions involved secretory pathways (MIF, RESISTIN, CypA), cell-cell contact (CD99, APP), and extracellular matrix receptor interactions (FN1), through key ligand-receptor (LR) pairs such as *RETN:CAP1*, *FN1*:*CD44*, *MIF*:(*CD74*+*CD44*), and *CD99*: *CD99* (**Figure 6C**).

Second, neutrophils acted as primary signal recipients, prominently involved in the MHC-II signaling pathway (**Figure 6A**, black dashed lines, **Supplementary Figure 10B**). In this context, *CD4* was predicted to function as a key receptor, with *HLA*-*DRB1*^+^ subsets serving as ligand-bearing partners (**Figure 6D**). Third, effector immune subsets, including *CD4*^+^ T cells, *CD8*^+^ T cells, and plasma cells (clusters 0, 1, 2, 4, 5, 15, 16, 22, 24, 28, 29, 31, and 32), were predominantly engaged in MIF and to a lesser extent CD99 signaling. These involved the LR pairs MIF:(CD74+CD44) and CD99:CD99 (**Figures 6A**, blue dashed lines, **6E**, **Supplementary Figures 10C, 10D**). Lastly, naïve and DN lymphocytes participated moderately in cell-cell contact (via SELPLG, LCK, and MHC-II) and secretory (GALECTIN, MIF) signaling pathways. Key LR genes included *SELL*, *CD44*, *CD74*, and *CD99* (**Figures 6A, F**, **Supplementary Figure 10A**). All identified LR genes are listed in **Figure 6G**. These interactome analyses highlight key ligand-receptor axes and signaling pathways critical for immune crosstalk and homeostasis in healthy dogs.

### Integrated scRNA-seq analysis of circulating leukocytes in healthy dogs

Healthy dogs immunologically experience diverse and continuous antigenic stimulation (58), exhibiting a broad and dynamic immune repertoire shaped by environmental exposures (9, 59, 60). To explore cohort-specific immune variation, we performed an integrated analysis of publicly available scRNA-seq datasets of peripheral leukocytes from two independent cohorts of healthy dogs (8, 9). Using a standardized pipeline for preprocessing, quality control, data integration, and removal of potential doublets and dataset-origin bias (**Supplementary Figure 11A**), we analyzed 90,992 canine immune cells, ultimately identifying 35 transcriptionally distinct clusters in the integrated Seurat object (**Supplementary Figure 11B**). Cluster-defining marker genes are listed in **Supplementary Data 19**.

Integration revealed that immune cells from both cohorts generally retained a shared transcriptomic structure (**Supplementary Figure 11C**). However, proportional differences in immune subsets were observed between cohorts (**Supplementary Figure 11D**), such as clusters 16, 29, and 33 characterized by gene expression associated with platelets (*PPBP*), erythrocytes (*LOC100855558*, a hemoglobin subunit alpha-like gene), and B cells (*CD79B*), respectively (**Supplementary Figure 11E**). One particularly study-specific subset, cluster 25, exhibited high expression of cell cycle-related genes (*PCLAF*, *MKI67*, and *SPC24*) and was enriched for a T cell cycling signature (**Supplementary Figures 11F, G**), suggesting a population of proliferating T cells potentially responding to steady-state antigenic stimuli (57). We further examined cohort-dependent expression of IC genes. Across studies, the proportions of *CTLA4*^+^ and *LAG3*^+^ T cells significantly differed (**Supplementary Figures 11H, I**, **Table 1**). Consistent with prior observations (61), *CTLA4*^+^ T cells comprised approximately 25% of circulating T cells in healthy dogs. Additionally, proportions of *PDCD1*^+^, *HAVCR2*^+^, *TIGIT*^+^ T cells, and *CD274*^+^ myeloid cells differed by cohort. For example, Ammons et al. (8) rarely observed *PDCD1*^+^ T cells in the cohort; however, *PDCD1*^+^ T cells represented 4.1% of the overall T cell count in the cohort by Eschke et al. (9). These *PDCD1*^+^ T cells, associated with IC gene expression, were linked to immune activation, whereas *LAG3*^+^ and *TIM3*^+^*PDCD1*^+^ T cells were enriched for T cell exhaustion signatures (**Supplementary Figure 11J**). Importantly, T cell exhaustion enrichment scores differed significantly between studies (**Supplementary Figure 11K**), highlighting distinct immunological imprints shaped by environment, lifestyle, or microbial exposure. In summary, this integrated scRNA-seq analysis uncovers cohort-specific immune features in healthy dogs, providing evidence on how environmental context contributes to immune diversity and checkpoint-related immune phenotypes.

**Table 1:** Proportions of immune subset with IC genes across cohorts.

Cell type	Genes	Proportion of immune subset in this study (%)	Ammons et al., 2023	Escheke et al., 2023
T cells	*PDCD1*	5.7 ± 2.5	0.2 ± 0.1	4.1 ± 1.6
	*CTLA4*	15.9 ± 3.8	25.2 ± 5.0	32.3 ± 6.2
	*LAG3*	3.4 ± 0.8	2.5 ± 1.0	6.4 ± 2.6
	*TIGIT*	0.7 ± 0.6	1.7 ± 0.3	1.3 ± 0.4
	*HAVCR2*	1.4 ± 0.7	3.1 ± 1.6	1.9 ± 0.3
	*CD274*	0.7 ± 0.4	1.5 ± 0.6	1.5 ± 0.5
Myeloid cells	*CD274*	1.8 ± 1.7	5.2 ± 3.2	

## Discussion

Companion dogs are recognized as valuable translational animal models for studying human diseases. Although emerging studies have applied scRNA-seq to canine peripheral leukocytes (8–10, 13, 16, 20, 23), the identification of functionally distinct and clinically relevant immune subsets, along with the elucidation of their roles and underlying molecular mechanisms in immune homeostasis, remains largely unexplored. To address the existing knowledge gap, we constructed a single-cell atlas of peripheral leukocytes from clinically healthy dogs adopting the canFam4 genome reference for scRNA-seq analysis for the first time. We demonstrate that the canFam4 provides a unique advantage over canFam3.1 by improving single-cell recovery, enabling the discovery of previously uncharacterized immune subsets with potential clinical relevance. To our knowledge, this is the first study to outline transcriptional signatures associated with IC genes, such as *PDCD1*, *CD274*, and *LAG3*, in healthy dogs, providing initial insights into their potential roles in normal immune regulation. We also delineated core transcriptional features of normal B cell differentiation, providing additional reference points for future immunological and clinical interpretations. Finally, we demonstrate that environmental context has great potential to shape cohort-dependent immune phenotypes and diversity. Collectively, our dataset and analytical framework offer a valuable resource for translational immunology in dogs, with implications for cancer research and beyond.

Notably, canFam3.1 has traditionally served as the primary reference for canine transcriptomics; however, recent studies have begun to adopt canFam4 owing to its superior contiguity and improved transcriptional resolution (30, 62, 63). In our dataset, canFam4 increased the number of assignable cells and reduced reads mapping to non-canine homologs, while enabling detection of previously unannotated gene features. These advantages allowed clearer delineation of transcriptionally heterogeneous peripheral immune subsets in dogs. For example, *CD14*^+^ monocytes have been proposed to resemble *CD4*^+^ monocytes in dogs (8), yet our data showed minimal *CD4* expression in *CD14*^+^ monocytes, with *CD4* instead primarily detected in neutrophils, consistent with prior flow cytometric findings (64). Although CD16 mRNA and protein are clearly detectable in canine immune cells (43, 65), the *CD16* gene itself is not annotated in the current canFam4 genome assembly. Consequently, classification of *CD16*^+^ subsets, such as NK cells and *CD14*^+^*CD16*^+^ monocytes, using scRNA-seq mapped to either canFam4 or 3.1 may be limited, meaning the related functional pathway interpretations should, therefore, be approached with caution. In addition to known markers such as *CD1c*, *CD86*, *CD83*, and *IL3RA*, we also identified features including *CD11c* (*ITGAX*), CD1 family genes (*CD1A*, *CD1B*, *CD1D*, *CD1E*), *HLA*- *DRB1*, *DLA*-*DQB1*, *DLA*-*64*, and *CD33*, which may refine annotation of DC and granulocyte/MDSC-like subsets in dogs. Likewise, genes such as *CD21*, *CD24*, and *CEACAM1* may aid in identifying low-density granulocytes or MDSC-like populations. For T cells, *GLNY*, *NKG7*, *GZMH*, *IL32*, and *BATF* may improve delineation of effector *CD8*^+^ T cells and *CD4*^+^ Treg subsets.

Given that current canine genome references remain incompletely annotated (10), updated assemblies, such as canFam4, may be essential for uncovering the clinical relevance of previously uncharacterized immune subsets (8, 10, 66) and for improving T cell annotation in single-cell datasets. Notably, IL32 and BATF have been implicated in the immunosuppressive functions of tumor-infiltrating Tregs (67, 68), suggesting that IL32^+^BATF^+^ Tregs in dogs may represent a regulatory subset relevant in cancer. Similarly, CEACAM1 and CD24 associated with neutrophil-mediated immunosuppression and T cell tolerance in other species (69–71) were detected in canine granulocytes (72). These findings highlight the value of canFam4-based profiling in identifying immune regulatory programs and suggest that CEACAM1^+^CD24^+^ granulocytes may warrant further investigation in canine models of infection, inflammation, and cancer. By focusing on *CD14*^+^ monocytes, we further identified a previously unrecognized *S100A4*^+^*CCL23*^+^*TNFSF13*^+^ subset, which may function as tissue-recruiting and matrix-modifying cells rather than classical cytokine-driven inflammatory effectors. Finally, we report the presence of peripheral XCR1^+^ DCs in dogs consistent with findings in humans, mice, and cattle (73–75), which highlights the conserved cDC1 biology across species. Together, these insights underscore the utility of updated genome assemblies, such as canFam4, for revealing clinically and mechanistically relevant immune subsets in canine immunology and translational research (8, 10, 66).

Several immune subsets, including *CD14*^+^ monocytes, *CD4*^+^ T cells, and B cells, displayed type I IFN-associated transcriptional programs, with all three populations sharing MMVD-related IFN signatures. These findings indicate a conserved IFN-driven activation axis, suggesting that these subsets might represent a pre-activated immune landscape under steady-state conditions. Similar IFN pathway activation has been reported in canine MMVD, where peripheral leukocytes and valvular tissues exhibit elevated *MX1* and *ISG15* expression (76, 77). Comparative analysis with published canine datasets confirmed *MX1* upregulation in MMVD-affected valves (77) and downregulation of related ISGs in early-stage disease (45), consistent with IFN-mediated antiviral and tissue-protective responses also described in human cardiac tissue (78). IFN-enriched *CD4*^+^ T and *CD14*^+^ monocyte subsets, previously characterized in dogs as tissue-patrolling or inflammatory responders (8, 22, 24), may constitute a surveillance network capable of rapid activation under stress or infection. In humans, immune subsets with constitutive expression of IFN-stimulated genes, such as *MX1*, are considered pre-activated population that can mount rapid responses to malignant cells, pathogens, or autoantigens (31, 79–81). Although the specific role of IFN-related B cells in canine MMVD remains unclear, their transcriptional similarity to type I IFN and MMVD signatures may suggest possible immunological relevance, potentially reflecting a steady-state antiviral-primed B cell state emerging along one of the early activation trajectories. Meanwhile, the *CD177*^+^ neutrophil subset also overlapped transcriptionally with neutrophil-enriched MMVD and IMHA datasets in dogs (36, 76), both characterized by *MMP9* and *PTX3* upregulation involved in tissue remodeling and immune activation. Given that our dataset derives from healthy dogs, these shared transcriptional patterns are more plausibly interpreted as primed innate immune phenotypes rather than active pathology. Collectively, our results suggest that IFN-related and *CD177*^+^ subsets represent pre-activated immune states with transcriptomic overlap to canine MMVD and IMHA profiles. Future validation using disease-cohort single-cell and proteomic datasets will be essential to clarify whether these subsets act as early mediators of immune dysregulation or serve as components of steady-state immune surveillance in dogs.

As in humans, many naturally occurring cancers in dogs exhibit features of immunogenicity, although the magnitude and characteristics of these responses vary across tumor types (82, 83). Recent clinical studies have shown that targeting immune-checkpoint pathways in dogs is feasible and biologically active, and cross-species evidence indicates that canine PD1/PD-L1 signaling is structurally and functionally comparable to that of humans (46, 47, 56, 84–86). Consistent with this, the fundamental inhibitory roles of PD1 and PD-L1 are well established in humans, including PD1-mediated regulation of T cell immunity (87) and PD-L1-induced T cell apoptosis as a tumor immune evasion mechanism (88). However, despite these well-characterized pathways in humans, the canine-specific biological roles and regulatory mechanisms of key checkpoint genes, such as *PDCD1*, *CD274*, *LAG3*, and *CTLA4*, remain comparatively underexplored. In this study, we leveraged scRNA-seq to begin addressing this gap in the canine immune system. First, consistent with a previous finding (53), our data show that PDCD1 exerts inhibitory effects on canine T cells. Although activation-associated genes were upregulated in *PDCD1*^+^*CD4*^+^ and *CD8*^+^ T cells, several TCR signaling genes (*ID3*, *CCR7*, *FOXP1*, *TRAT1*, *IL7R*) were downregulated. These results support the notion that *PDCD1* can reflect both activation and early exhaustion; however, in healthy dogs, *PDCD1* expression most likely indicates recent activation, with exhaustion becoming relevant only under chronic inflammatory contexts when additional inhibitory receptors co-occur. Second, *LAG3* contributed to exhaustion-related programs, particularly in *LAG3*^+^*PDCD1*^+^*CD8*^+^ T cells, which showed reduced *TBX21* expression, consistent with human data indicating that T-bet represses LAG3 to maintain functional PD1^+^CD8^+^ T cell responses (89, 90). The transcriptional signatures of these cells were also observed within the tumor microenvironment of canine osteosarcoma, suggesting that similar regulatory mechanisms may influence tumor-associated T cell dysfunction. Additionally, *LAG3* expression correlated with upregulation of MDH1 and MDH2, suggesting that malate metabolism plays a role in sustaining PD1^+^CD4^+^ T cell function, as described previously in chronic viral infection models (91). Future studies are warranted to determine whether malate metabolism directly modulates the functional stability of CD4^+^ T cells and prevents the transition of these cells into exhaustion—a phenomenon that remains to be demonstrated in both humans and dogs. Third, we found evidence that *CD274*^+^ neutrophils and monocytes may contribute to IL-10-mediated immunosuppression. In dogs, IL-10 impairs neutrophil function during babesiosis (92), and in humans, PD-L1^+^ myeloid cells are major IL-10 producers in several cancers (93, 94). Therefore, our findings suggest that PD-L1^+^ neutrophils or PMN-MDSCs may also contribute to shaping an immunosuppressive microenvironment in dogs. Finally, consistent with previous reports (54, 61, 95), *CTLA4* expression was observed in regulatory T subsets and notably in DN T cells. Although DN T cells lacked canonical regulatory markers (*FOXP3*, *IL10*, *IL2RA*, *GATA3*) (96, 97), their *CTLA4* expression, naïve-like origin, and absence of exhaustion-related signals based on trajectory analysis suggest that *CTLA4* may act as an early regulatory cue guiding DN T cell differentiation. While direct evidence of DN T cells in canine tumors is limited, their immunoregulatory properties in tissues (97, 98) and the clinical activity of CTLA4-targeted therapies in dogs (54) support a potential functional role within the tumor microenvironment.

PBMCs reflect peripheral immunity and are influenced by diverse factors, including breed, lifestyle, and environmental exposures (99–101). In healthy dogs, circulating T and myeloid cells express PD1, PD-L1, and CTLA4 (54, 102, 103). Herein, we investigated the cohort-specific effects on PBMC immune profiles by integrating publicly available scRNA-seq datasets from healthy dogs. For the first time, our integrated analysis delineated distinct cohort-dependent immune features, reflected by differences in the proportions of cycling T cells, checkpoint-expressing subsets, and T cell exhaustion states. In dogs, cycling T cells have been detected in the periphery, duodenum, Peyer’s patches, and mesenteric lymph nodes, indicating ongoing antigenic stimulation (8, 14, 15, 22). This is further supported by our interactome analysis, which reveals that cycling T cells interact with *CD14*^+^ monocytes, DC, or pDC mediated through MIF and CD99 signaling pathways. Additionally, each cohort exhibited distinct patterns of IC gene expression, such as *CTLA4* and *LAG3*, along with variable of T cell exhaustion states, underscoring the immune heterogeneity among individuals. These differences likely reflect cohort-specific antigen exposure. PBMC datasets have served as essential resources for studies of naturally occurring diseases (8, 104, 105) and for investigating the impacts of environmental and lifestyle factors on immunity (106–109). Accordingly, it may be preferable to adopt immune cell reference datasets that are tailored to the local context, such as domestic conditions in South Korea, and the specific research purpose.

Finally, we investigated homeostatic cell–cell interactions among peripheral immune subsets in healthy dogs. Our interactome analysis indicated that MIF, CD99, MHC-II, and SELPLG constitute key intrinsic pathways governing immune homeostasis. Interestingly, Moore et al. (110) previously proposed that CD4 expressed on canine neutrophils interacts with receptors expressed on leukocytes. Consistent with this model, our analysis predicted interactions between *CD4* on neutrophils and *HLA*- *DRB1* on antigen-presenting cells. Notably, human and canine neutrophils endogenously express surface CD4 (111), which can bind certain viral proteins that enhance inflammatory responses. CD4 expression has also been associated with increased neutrophil migratory capacity (112). In agreement with these findings, *CD4*^+^ neutrophils in our dataset were strongly enriched in inflammatory transmigration pathways, suggesting that MHC-II-mediated interactions may regulate the biodistribution of these cells *in vivo*. Furthermore, CD99 and MIF signaling emerged as key components of myeloid-myeloid communication. Canine macrophages infiltrating local or metastatic oral melanoma have been reported to significantly upregulate both molecules compared with normal mucosal tissues (113). Meanwhile, elevated circulating levels have also been described in atopic dermatitis and mammary neoplasia (114, 115). In our analysis, *CD14*^+^ monocytes and DCs displayed homotypic interactions through CD99 and MIF, and *XCR1*^+^ DCs showed specific expression of MIF. Similar homotypic myeloid interactions have been implicated in shaping anti-tumor immunity in human melanoma patients (116). Collectively, these findings suggest that CD99- and MIF-mediated signaling may represent important components of canine immune homeostasis that may extend beyond the steady state to inflammatory or tumor-associated contexts. These predictions warrant experimental validation using single-cell and spatial transcriptomic approaches.

We have several limitations to acknowledge. First, the sample size was small, limiting representation of the full biological diversity of companion dogs. This includes potential variation associated with breed, age, and individual immune histories, which could not be fully assessed in our cohort. Second, we intentionally enriched CD3^+^ T cells to ensure adequate representation of lymphocyte populations, which may not fully reflect the physiological leukocyte distribution in peripheral blood. However, this approach would provide a more appropriate reference for future T cell-focused studies, particularly in contexts where stress leukograms with neutrophilia and lymphopenia are marked. Third, as with all scRNA-seq studies, our conclusions are based on transcriptional profiles rather than functional assays, and computational inferences, such as module scoring or trajectory analysis, cannot fully substitute for direct experimental validation. In addition, despite using the most recent canFam4 assembly, incomplete annotation of canine genes still poses inherent limitations for functional interpretation. Continued efforts to further refine the canine genome and its transcriptomic annotation, similar to the mature resources available for human and mouse studies, will greatly enhance future immunological and comparative transcriptomic research.

In conclusion, to our knowledge, this is the first study to map the single-cell transcriptomic landscape of circulating leukocytes in South Korean dogs. Our results provide a valuable resource to support future scRNA-seq studies on immune cells in dogs affected by various immunological disorders. Methodology used in this study may help pave the way for investigating potential roles of IC genes in translational research, bridging canine and human immunology.

## Data Availability

The datasets presented in this study can be found in online repositories. The names of the repository/repositories and accession number(s) can be found in the article/**Supplementary Material**.

## References

[1] Reif JS . Animal sentinels for environmental and public health. Public Health Rep. (2011) 126 Suppl 1:50–7. doi: 10.1177/00333549111260S108, PMID: 21563712 PMC3072903

[2] Kol A Arzi B Athanasiou KA Farmer DL Nolta JA Rebhun RB . Companion animals: Translational scientist’s new best friends. Sci Transl Med. (2015) 7:308ps21. doi: 10.1126/scitranslmed.aaa9116, PMID: 26446953 PMC4806851

[3] Arnold C . Sick as a dog: how understanding canine diseases will save human lives. Nat Med. (2022) 28:1970–3. doi: 10.1038/s41591-022-02025-5, PMID: 36180595

[4] Vázquez-Baeza Y Hyde ER Suchodolski JS Knight R . Dog and human inflammatory bowel disease rely on overlapping yet distinct dysbiosis networks. Nat Microbiol. (2016) 1:16177. doi: 10.1038/nmicrobiol.2016.177, PMID: 27694806

[5] Momozawa Y . The potential of translational research in dogs in human medicine. Trans Regul Sci. (2019) 1:31–6. doi: 10.33611/trs.1_31

[6] LeBlanc AK Mazcko CN . Improving human cancer therapy through the evaluation of pet dogs. Nat Rev Cancer. (2020) 20:727–42. doi: 10.1038/s41568-020-0297-3, PMID: 32934365

[7] Jovic D Liang X Zeng H Lin L Xu F Luo Y . Single-cell RNA sequencing technologies and applications: A brief overview. Clin Transl Med. (2022) 12:e694. doi: 10.1002/ctm2.694, PMID: 35352511 PMC8964935

[8] Ammons DT Harris RA Hopkins LS Kurihara J Weishaar K Dow S . A single-cell RNA sequencing atlas of circulating leukocytes from healthy and osteosarcoma affected dogs. Front Immunol. (2023) 14:1162700. doi: 10.3389/fimmu.2023.1162700, PMID: 37275879 PMC10235626

[9] Eschke M Moore PF Chang H Alber G Keller SM . Canine peripheral blood TCRαβ T cell atlas: Identification of diverse subsets including CD8A+ MAIT-like cells by combined single-cell transcriptome and V(D)J repertoire analysis. Front Immunol. (2023) 14:1123366. doi: 10.3389/fimmu.2023.1123366, PMID: 36911660 PMC9995359

[10] Hoang MH Skidmore ZL Rindt H Chu S Fisk B Foltz JA . Single-cell T-cell receptor repertoire profiling in dogs. Commun Biol. (2024) 7:484. doi: 10.1038/s42003-024-06174-w, PMID: 38649520 PMC11035579

[11] Jackson K Milner RJ Doty A Hutchison S Cortes-Hinojosa G Riva A . Analysis of canine myeloid-derived suppressor cells (MDSCs) utilizing fluorescence-activated cell sorting, RNA protection mediums to yield quality RNA for single-cell RNA sequencing. Vet Immunol Immunopathol. (2021) 231:110144. doi: 10.1016/j.vetimm.2020.110144, PMID: 33278779

[12] Razmara AM Farley LE Harris RM Judge SJ Lammers M Iranpur KR . Preclinical evaluation and first-in-dog clinical trials of PBMC-expanded natural killer cells for adoptive immunotherapy in dogs with cancer. J Immunother Cancer. (2024) 12:e007963. doi: 10.1136/jitc-2023-007963, PMID: 38631708 PMC11029326

[13] Lee K-H Lee D Lee J-W Hwang H-J Cho J-Y . Transcriptomic profiling of PBMCs from mammary tumor dogs reveals two distinct immune states. Am J Cancer Res. (2025) 15:2564–78. doi: 10.62347/LOJB9067, PMID: 40667535 PMC12256404

[14] Miguelena Chamorro B Hameed SA Dechelette M Claude J-B Piney L Chapat L . Characterization of canine peyer’s patches by multidimensional analysis: insights from immunofluorescence, flow cytometry, and single-cell RNA sequencing. Immunohorizons. (2023) 7:788–805. doi: 10.4049/immunohorizons.2300091, PMID: 38015460 PMC10696420

[15] Chamorro BM Hameed SA Claude J-B Piney L Chapat L Swaminathan G . Canine mesenteric lymph nodes (MLNs) characterization by sc-RNAseq: insights compared to human and mouse MLNs. Sci Rep. (2024) 14:20290. doi: 10.1038/s41598-024-71310-9, PMID: 39217215 PMC11365970

[16] Razmara AM Lammers M Judge SJ Murphy WJ Gaskill CE Culp WTN . Single cell atlas of canine natural killer cells identifies distinct circulating and tissue resident gene profiles. Front Immunol. (2025) 16:1571085. doi: 10.3389/fimmu.2025.1571085, PMID: 40443661 PMC12119461

[17] Fastrès A Pirottin D Fievez L Marichal T Desmet CJ Bureau F . Characterization of the bronchoalveolar lavage fluid by single cell gene expression analysis in healthy dogs: A promising technique. Front Immunol. (2020) 11:1707. doi: 10.3389/fimmu.2020.01707, PMID: 32849601 PMC7406785

[18] Zhou Q-J Liu X Zhang L Wang R Yin T Li X . A single-nucleus transcriptomic atlas of the dog hippocampus reveals the potential relationship between specific cell types and domestication. Natl Sci Rev. (2022) 9:nwac147. doi: 10.1093/nsr/nwac147, PMID: 36569494 PMC9772819

[19] Rizzoli E Fievez L Fastrès A Roels E Marichal T Clercx C . A single-cell RNA sequencing atlas of the healthy canine lung: a foundation for comparative studies. Front Immunol. (2025) 16:1501603. doi: 10.3389/fimmu.2025.1501603, PMID: 40114924 PMC11922831

[20] Li B Ding Y Han M Zhang Z Ling Z Zhu W . Multi-omics analysis of canine aging markers and evaluation of stem cell intervention. Commun Biol. (2025) 8:905. doi: 10.1038/s42003-025-08333-z, PMID: 40494876 PMC12152182

[21] Fastrès A Pirottin D Fievez L Tutunaru A-C Bolen G Merveille A-C . Identification of pro-fibrotic macrophage populations by single-cell transcriptomic analysis in west highland white terriers affected with canine idiopathic pulmonary fibrosis. Front Immunol. (2020) 11:611749. doi: 10.3389/fimmu.2020.611749, PMID: 33384697 PMC7770158

[22] Manchester AC Ammons DT Lappin MR Dow S . Single cell transcriptomic analysis of the canine duodenum in chronic inflammatory enteropathy and health. Front Immunol. (2024) 15:1397590. doi: 10.3389/fimmu.2024.1397590, PMID: 38933260 PMC11199541

[23] Früh SP Saikia M Eule J Mazulis CA Miller JE Cowulich JM . Elevated circulating Th2 but not group 2 innate lymphoid cell responses characterize canine atopic dermatitis. Vet Immunol Immunopathol. (2020) 221:110015. doi: 10.1016/j.vetimm.2020.110015, PMID: 32058160

[24] Ammons DT Hopkins LS Cronise KE Kurihara J Regan DP Dow S . Single-cell RNA sequencing reveals the cellular and molecular heterogeneity of treatment-naïve primary osteosarcoma in dogs. Commun Biol. (2024) 7:496. doi: 10.1038/s42003-024-06182-w, PMID: 38658617 PMC11043452

[25] Cambien B Lebrigand K Baeri A Nottet N Compin C Lamit A . Identification of oncolytic vaccinia restriction factors in canine high-grade mammary tumor cells using single-cell transcriptomics. PloS Pathog. (2020) 16:e1008660. doi: 10.1371/journal.ppat.1008660, PMID: 33075093 PMC7595618

[26] Ayers J Milner RJ Cortés-Hinojosa G Riva A Bechtel S Sahay B . Novel application of single-cell next-generation sequencing for determination of intratumoral heterogeneity of canine osteosarcoma cell lines. J Vet Diagn Invest. (2021) 33:261–78. doi: 10.1177/1040638720985242, PMID: 33446089 PMC7944434

[27] Wallace MD Herrtage ME Gostelow R Owen L Rutherford L Hughes K . Single-cell transcriptomic analysis of canine insulinoma reveals distinct sub-populations of insulin-expressing cancer cells. Vet Oncol. (2025) 2:13. doi: 10.1186/s44356-025-00026-3, PMID: 40438247 PMC12106163

[28] Ammons DT Contursi C Olsen M Yoshimoto JA Owens E Harris M . Transcriptional landscape of canine hematopoiesis and cross-species comparisons revealed by single-cell RNA sequencing. Res Sq. (2025). doi: 10.21203/rs.3.rs-6299609/v1

[29] Wang M Liao C Wu D Geng N Xia Y Chen Y . Single-Cell Transcriptome Atlas of the Canine Peri-implantitis Reveals stromal–immune cells interaction. Res Sq. (2024). doi: 10.21203/rs.3.rs-5348271/v1

[30] Wang C Wallerman O Arendt M-L Sundström E Karlsson Å Nordin J . A novel canine reference genome resolves genomic architecture and uncovers transcript complexity. Commun Biol. (2021) 4:185. doi: 10.1038/s42003-021-01698-x, PMID: 33568770 PMC7875987

[31] Kim M-C De U Borcherding N Wang L Paek J Bhattacharyya I . Single-cell transcriptomics unveil profiles and interplay of immune subsets in rare autoimmune childhood Sjögren’s disease. Commun Biol. (2024) 7:481. doi: 10.1038/s42003-024-06124-6, PMID: 38641668 PMC11031574

[32] Kim M-C Borcherding N Ahmed KK Voigt AP Vishwakarma A Kolb R . CD177 modulates the function and homeostasis of tumor-infiltrating regulatory T cells. Nat Commun. (2021) 12:5764. doi: 10.1038/s41467-021-26091-4, PMID: 34599187 PMC8486774

[33] Wen W Su W Tang H Le W Zhang X Zheng Y . Immune cell profiling of COVID-19 patients in the recovery stage by single-cell sequencing. Cell Discov. (2020) 6:31. doi: 10.1038/s41421-020-0168-9, PMID: 32377375 PMC7197635

[34] Aran D Looney AP Liu L Wu E Fong V Hsu A . Reference-based analysis of lung single-cell sequencing reveals a transitional profibrotic macrophage. Nat Immunol. (2019) 20:163–72. doi: 10.1038/s41590-018-0276-y, PMID: 30643263 PMC6340744

[35] Borcherding N Vishwakarma A Voigt AP Bellizzi A Kaplan J Nepple K . Mapping the immune environment in clear cell renal carcinoma by single-cell genomics. Commun Biol. (2021) 4:122. doi: 10.1038/s42003-020-01625-6, PMID: 33504936 PMC7840906

[36] Borchert C Herman A Roth M Brooks AC Friedenberg SG . RNA sequencing of whole blood in dogs with primary immune-mediated hemolytic anemia (IMHA) reveals novel insights into disease pathogenesis. PloS One. (2020) 15:e0240975. doi: 10.1371/journal.pone.0240975, PMID: 33091028 PMC7580939

[37] Sugawara-Suda M Morishita K Ichii O Namba T Aoshima K Kagawa Y . Transcriptome and proteome analysis of dogs with precursor targeted immune-mediated anemia treated with splenectomy. PloS One. (2023) 18:e0285415. doi: 10.1371/journal.pone.0285415, PMID: 37146011 PMC10162568

[38] Lee K-H Park H-M Son K-H Shin T-J Cho J-Y . Transcriptome signatures of canine mammary gland tumors and its comparison to human breast cancers. Cancers (Basel). (2018) 10. doi: 10.3390/cancers10090317, PMID: 30205506 PMC6162473

[39] Passos Barbosa MM Kamerer RL Schmit J Lopez AJ Uyehara R Tighe R . Preclinical evaluation of an anchored immunotherapy strategy with aluminum hydroxide-tethered IL-12 in dogs with advanced Malignant melanoma. Mol Cancer Ther. (2025) 24:406–18. doi: 10.1158/1535-7163.MCT-24-0317, PMID: 39632727 PMC11879767

[40] Stinson JA Barbosa MMP Sheen A Momin N Fink E Hampel J . Tumor-localized interleukin-2 and interleukin-12 combine with radiation therapy to safely potentiate regression of advanced Malignant melanoma in pet dogs. Clin Cancer Res. (2024) 30:4029–43. doi: 10.1158/1078-0432.CCR-24-0861, PMID: 38980919 PMC11398984

[41] Jin S Guerrero-Juarez CF Zhang L Chang I Ramos R Kuan C-H . Inference and analysis of cell-cell communication using CellChat. Nat Commun. (2021) 12:1088. doi: 10.1038/s41467-021-21246-9, PMID: 33597522 PMC7889871

[42] Li Z Sun C Wang F Wang X Zhu J Luo L . Molecular mechanisms governing circulating immune cell heterogeneity across different species revealed by single-cell sequencing. Clin Transl Med. (2022) 12:e689. doi: 10.1002/ctm2.689, PMID: 35092700 PMC8800483

[43] Hullsiek R Li Y Snyder KM Wang S Di D Borgatti A . Examination of igg fc receptor CD16A and CD64 expression by canine leukocytes and their ADCC activity in engineered NK cells. Front Immunol. (2022) 13:841859. doi: 10.3389/fimmu.2022.841859, PMID: 35281028 PMC8907477

[44] Heger L Hatscher L Liang C Lehmann CHK Amon L Lühr JJ . XCR1 expression distinguishes human conventional dendritic cell type 1 with full effector functions from their immediate precursors. Proc Natl Acad Sci USA. (2023) 120:e2300343120. doi: 10.1073/pnas.2300343120, PMID: 37566635 PMC10438835

[45] Kim T-S Hong C-Y Oh S-J Choe Y-H Hwang T-S Kim J . RNA sequencing provides novel insights into the pathogenesis of naturally occurring myxomatous mitral valve disease stage B1 in beagle dogs. PloS One. (2024) 19:e0300813. doi: 10.1371/journal.pone.0300813, PMID: 38753730 PMC11098313

[46] Maekawa N Konnai S Takagi S Kagawa Y Okagawa T Nishimori A . A canine chimeric monoclonal antibody targeting PD-L1 and its clinical efficacy in canine oral Malignant melanoma or undifferentiated sarcoma. Sci Rep. (2017) 7:8951. doi: 10.1038/s41598-017-09444-2, PMID: 28827658 PMC5567082

[47] Maekawa N Konnai S Nishimura M Kagawa Y Takagi S Hosoya K . PD-L1 immunohistochemistry for canine cancers and clinical benefit of anti-PD-L1 antibody in dogs with pulmonary metastatic oral Malignant melanoma. NPJ Precis Oncol. (2021) 5:10. doi: 10.1038/s41698-021-00147-6, PMID: 33580183 PMC7881100

[48] Goulart MR Pluhar GE Ohlfest JR . Identification of myeloid derived suppressor cells in dogs with naturally occurring cancer. PloS One. (2012) 7:e33274. doi: 10.1371/journal.pone.0033274, PMID: 22428007 PMC3302813

[49] Lang HP Osum KC Friedenberg SG . A review of CD4+ T cell differentiation and diversity in dogs. Vet Immunol Immunopathol. (2024) 275:110816. doi: 10.1016/j.vetimm.2024.110816, PMID: 39173398 PMC11421293

[50] Wu Y Chang Y-M Stell AJ Priestnall SL Sharma E Goulart MR . Phenotypic characterisation of regulatory T cells in dogs reveals signature transcripts conserved in humans and mice. Sci Rep. (2019) 9:13478. doi: 10.1038/s41598-019-50065-8, PMID: 31530890 PMC6748983

[51] Maeda S Motegi T Iio A Kaji K Goto-Koshino Y Eto S . Anti-CCR4 treatment depletes regulatory T cells and leads to clinical activity in a canine model of advanced prostate cancer. J Immunother Cancer. (2022) 10. doi: 10.1136/jitc-2021-003731, PMID: 35131860 PMC8804701

[52] Maeda S Murakami K Inoue A Yonezawa T Matsuki N . CCR4 blockade depletes regulatory T cells and prolongs survival in a canine model of bladder cancer. Cancer Immunol Res. (2019) 7:1175–87. doi: 10.1158/2326-6066.CIR-18-0751, PMID: 31160277

[53] Coy J Caldwell A Chow L Guth A Dow S . PD-1 expression by canine T cells and functional effects of PD-1 blockade. Vet Comp Oncol. (2017) 15:1487–502. doi: 10.1111/vco.12294, PMID: 28120417

[54] Maekawa N Konnai S Watari K Takeuchi H Nakanishi T Tachibana T . Development of caninized anti-CTLA-4 antibody as salvage combination therapy for anti-PD-L1 refractory tumors in dogs. Front Immunol. (2025) 16:1570717. doi: 10.3389/fimmu.2025.1570717, PMID: 40463388 PMC12130249

[55] Liu Y Huang J Pandey R Liu P Therani B Qiu Q . Robustness of single-cell RNA-seq for identifying differentially expressed genes. BMC Genomics. (2023) 24:371. doi: 10.1186/s12864-023-09487-y, PMID: 37394518 PMC10316566

[56] Igase M Inanaga S Nishibori S Itamoto K Sunahara H Nemoto Y . Proof-of-concept study of the caninized anti-canine programmed death 1 antibody in dogs with advanced non-oral Malignant melanoma solid tumors. J Vet Sci. (2024) 25:e15. doi: 10.4142/jvs.23144, PMID: 38311328 PMC10839171

[57] Li W-P Mao X-T Xie J-H Li J-Y Liu B-Q Wu L-X . N-acetyltransferase 10 is implicated in the pathogenesis of cycling T cell-mediated autoimmune and inflammatory disorders in mice. Nat Commun. (2024) 15:9388. doi: 10.1038/s41467-024-53350-x, PMID: 39477944 PMC11525920

[58] Dow S . A role for dogs in advancing cancer immunotherapy research. Front Immunol. (2019) 10:2935. doi: 10.3389/fimmu.2019.02935, PMID: 32010120 PMC6979257

[59] Sexton C Ruple A . Canine sentinels and our shared exposome. Science. (2024) 384:1170–2. doi: 10.1126/science.adl0426, PMID: 38870288

[60] Hwang M-H Darzentas N Bienzle D Moore PF Morrison J Keller SM . Characterization of the canine immunoglobulin heavy chain repertoire by next generation sequencing. Vet Immunol Immunopathol. (2018) 202:181–90. doi: 10.1016/j.vetimm.2018.07.002, PMID: 30078594

[61] Marable J Ruiz D Jaiswal AK Bhattacharya R Pantazes R Agarwal P . Nanobody-based CTLA4 inhibitors for immune checkpoint blockade therapy of canine cancer patients. Sci Rep. (2021) 11:20763. doi: 10.1038/s41598-021-00325-3, PMID: 34675296 PMC8531395

[62] Mannheimer JD Tawa G Gerhold D Braisted J Sayers CM McEachron TA . Transcriptional profiling of canine osteosarcoma identifies prognostic gene expression signatures with translational value for humans. Commun Biol. (2023) 6:856. doi: 10.1038/s42003-023-05208-z, PMID: 37591946 PMC10435536

[63] Mason NJ Selmic L Ruple A London CA Barber L Weishaar K . Immunological responses and clinical outcomes in dogs with osteosarcoma receiving standard therapy and a Listeria vaccine expressing HER2. Mol Ther. (2025) 33:1674–86. doi: 10.1016/j.ymthe.2025.02.023, PMID: 39955616 PMC11997493

[64] McDonald E Kehoe E Deines D McCarthy M Wright B Huse S . High-parameter immunophenotyping reveals distinct immune cell profiles in pruritic dogs and cats. Front Vet Sci. (2024) 11:1498964. doi: 10.3389/fvets.2024.1498964, PMID: 39911485 PMC11795398

[65] Kim Y Lee S-H Kim C-J Lee J-J Yu D Ahn S . Canine non-B, non-T NK lymphocytes have a potential antibody-dependent cellular cytotoxicity function against antibody-coated tumor cells. BMC Vet Res. (2019) 15:339. doi: 10.1186/s12917-019-2068-5, PMID: 31610784 PMC6790994

[66] Hörtenhuber M Hytönen MK Mukarram AK Arumilli M Araujo CL Quintero I . The DoGA consortium expression atlas of promoters and genes in 100 canine tissues. Nat Commun. (2024) 15:9082. doi: 10.1038/s41467-024-52798-1, PMID: 39433728 PMC11494170

[67] Itahashi K Irie T Yuda J Kumagai S Tanegashima T Lin Y-T . BATF epigenetically and transcriptionally controls the activation program of regulatory T cells in human tumors. Sci Immunol. (2022) 7:eabk0957. doi: 10.1126/sciimmunol.abk0957, PMID: 36206353

[68] Han L Chen S Chen Z Zhou B Zheng Y Shen L . Interleukin 32 promotes foxp3+ treg cell development and CD8+ T cell function in human esophageal squamous cell carcinoma microenvironment. Front Cell Dev Biol. (2021) 9:704853. doi: 10.3389/fcell.2021.704853, PMID: 34414188 PMC8369465

[69] Zhao C Huang Y Zhang H Liu H . CD24 affects the immunosuppressive effect of tumor-infiltrating cells and tumor resistance in a variety of cancers. Discov Oncol. (2024) 15:399. doi: 10.1007/s12672-024-01284-7, PMID: 39222166 PMC11369128

[70] Pan H Shively JE . Carcinoembryonic antigen-related cell adhesion molecule-1 regulates granulopoiesis by inhibition of granulocyte colony-stimulating factor receptor. Immunity. (2010) 33:620–31. doi: 10.1016/j.immuni.2010.10.009, PMID: 21029969 PMC3765078

[71] Huang Y-H Zhu C Kondo Y Anderson AC Gandhi A Russell A . CEACAM1 regulates TIM-3-mediated tolerance and exhaustion. Nature. (2015) 517:386–90. doi: 10.1038/nature13848, PMID: 25363763 PMC4297519

[72] Kammerer R Popp T Härtle S Singer BB Zimmermann W . Species-specific evolution of immune receptor tyrosine based activation motif-containing CEACAM1-related immune receptors in the dog. BMC Evol Biol. (2007) 7:196. doi: 10.1186/1471-2148-7-196, PMID: 17945019 PMC2110893

[73] Domenjo-Vila E Casella V Iwabuchi R Fossum E Pedragosa M Castellví Q . XCR1+ DCs are critical for T cell-mediated immunotherapy of chronic viral infections. Cell Rep. (2023) 42:112123. doi: 10.1016/j.celrep.2023.112123, PMID: 36795562

[74] Crozat K Guiton R Contreras V Feuillet V Dutertre C-A Ventre E . The XC chemokine receptor 1 is a conserved selective marker of mammalian cells homologous to mouse CD8alpha+ dendritic cells. J Exp Med. (2010) 207:1283–92. doi: 10.1084/jem.20100223, PMID: 20479118 PMC2882835

[75] Li K Wei G Cao Y Li D Li P Zhang J . The Identification and Distribution of Cattle XCR1 and XCL1 among Peripheral Blood Cells: New Insights into the Design of Dendritic Cells Targeted Veterinary Vaccine. PloS One. (2017) 12:e0170575. doi: 10.1371/journal.pone.0170575, PMID: 28129380 PMC5271332

[76] Ljungvall I Rajamäki MM Crosara S Olsen LH Kvart C Borgarelli M . Evaluation of plasma activity of matrix metalloproteinase-2 and -9 in dogs with myxomatous mitral valve disease. Am J Vet Res. (2011) 72:1022–8. doi: 10.2460/ajvr.72.8.1022, PMID: 21801058

[77] Markby GR Macrae VE Summers KM Corcoran BM . Disease severity-associated gene expression in canine myxomatous mitral valve disease is dominated by TGFβ Signaling. Front Genet. (2020) 11:372. doi: 10.3389/fgene.2020.00372, PMID: 32395121 PMC7197751

[78] Zhou Q Pan L-L Xue R Ni G Duan Y Bai Y . The anti-microbial peptide LL-37/CRAMP levels are associated with acute heart failure and can attenuate cardiac dysfunction in multiple preclinical models of heart failure. Theranostics. (2020) 10:6167–81. doi: 10.7150/thno.46225, PMID: 32483446 PMC7255020

[79] Cooper L Xu H Polmear J Kealy L Szeto C Pang ES . Type I interferons induce an epigenetically distinct memory B cell subset in chronic viral infection. Immunity. (2024) 57:1037–1055.e6. doi: 10.1016/j.immuni.2024.03.016, PMID: 38593796 PMC11096045

[80] Wang X Shen X Chen S Liu H Hong N Zhong H . Reinvestigation of classic T cell subsets and identification of novel cell subpopulations by single-cell RNA sequencing. J Immunol. (2022) 208:396–406. doi: 10.4049/jimmunol.2100581, PMID: 34911770

[81] Yoon J Jang D Kim M-C Paek J Miller R Veronese B . Diminished SUV3 expression and its functional implications in the IFN-enriched monocyte subset of childhood Sjögren’s disease. Rheumatol (Oxford). (2025) 64:4393–403. doi: 10.1093/rheumatology/keaf193, PMID: 40268748 PMC12212910

[82] Joung Y Yoon J Lee DJ Song WJ Cheong J . A case of canine colorectal carcinoma *in situ* with regulatory T cell infiltration. J Vet Clin. (2024) 41:207–14. doi: 10.17555/jvc.2024.41.4.207

[83] Jeong S Yoon J Song WJ Cheong J Yun Y . A case of canine mammary comedocarcinoma with regulatory T cell infiltration. J Vet Clin. (2024) 41:215–22. doi: 10.17555/jvc.2024.41.4.215

[84] Igase M Hagimori K Inanaga S Mizoguchi H Itamoto K Sakurai M . The caninized anti-canine PD-1 monoclonal antibody in canine oral Malignant melanoma: Efficacy and exploratory biomarker analysis. BioRxiv. (2025). doi: 10.1101/2025.08.26.671889 PMC12829387 41571458

[85] Igase M Nemoto Y Itamoto K Tani K Nakaichi M Sakurai M . A pilot clinical study of the therapeutic antibody against canine PD-1 for advanced spontaneous cancers in dogs. Sci Rep. (2020) 10:18311. doi: 10.1038/s41598-020-75533-4, PMID: 33110170 PMC7591904

[86] Pantelyushin S Ranninger E Guerrera D Hutter G Maake C Markkanen E . Cross-reactivity and functionality of approved human immune checkpoint blockers in dogs. Cancers (Basel). (2021) 13. doi: 10.3390/cancers13040785, PMID: 33668625 PMC7918463

[87] Nishimura H Nose M Hiai H Minato N Honjo T . Development of lupus-like autoimmune diseases by disruption of the PD-1 gene encoding an ITIM motif-carrying immunoreceptor. Immunity. (1999) 11:141–51. doi: 10.1016/s1074-7613(00)80089-8, PMID: 10485649

[88] Dong H Strome SE Salomao DR Tamura H Hirano F Flies DB . Tumor-associated B7-H1 promotes T-cell apoptosis: a potential mechanism of immune evasion. Nat Med. (2002) 8:793–800. doi: 10.1038/nm730, PMID: 12091876

[89] Kao C Oestreich KJ Paley MA Crawford A Angelosanto JM Ali M-AA . Transcription factor T-bet represses expression of the inhibitory receptor PD-1 and sustains virus-specific CD8+ T cell responses during chronic infection. Nat Immunol. (2011) 12:663–71. doi: 10.1038/ni.2046, PMID: 21623380 PMC3306165

[90] Rudd CE Chanthong K Taylor A . Small molecule inhibition of GSK-3 specifically inhibits the transcription of inhibitory co-receptor LAG-3 for enhanced anti-tumor immunity. Cell Rep. (2020) 30:2075–2082.e4. doi: 10.1016/j.celrep.2020.01.076, PMID: 32075731

[91] Weisshaar N Ma S Ming Y Madi A Mieg A Hering M . The malate shuttle detoxifies ammonia in exhausted T cells by producing 2-ketoglutarate. Nat Immunol. (2023) 24:1921–32. doi: 10.1038/s41590-023-01636-5, PMID: 37813964 PMC10602850

[92] Celliers A Rautenbach Y Hooijberg E Christopher M Goddard A . Neutrophil myeloperoxidase index in dogs with babesiosis caused by babesia rossi. Front Vet Sci. (2020) 7:72. doi: 10.3389/fvets.2020.00072, PMID: 32133380 PMC7040022

[93] Zhang S Sun L Zuo J Feng D . Tumor associated neutrophils governs tumor progression through an IL-10/STAT3/PD-L1 feedback signaling loop in lung cancer. Transl Oncol. (2024) 40:101866. doi: 10.1016/j.tranon.2023.101866, PMID: 38128466 PMC10753083

[94] Asai A Yasuoka H Matsui M Tsuchimoto Y Fukunishi S Higuchi K . Programmed death 1 ligand expression in the monocytes of patients with hepatocellular carcinoma depends on tumor progression. Cancers (Basel). (2020) 12. doi: 10.3390/cancers12082286, PMID: 32824016 PMC7465257

[95] Mason NJ Chester N Xiong A Rotolo A Wu Y Yoshimoto S . Development of a fully canine anti-canine CTLA4 monoclonal antibody for comparative translational research in dogs with spontaneous tumors. MAbs. (2021) 13:2004638. doi: 10.1080/19420862.2021.2004638, PMID: 34856888 PMC8726733

[96] Protschka M Di Placido D Moore PF Büttner M Alber G Eschke M . Canine peripheral non-conventional TCRαβ+ CD4-CD8α- double-negative T cells show T helper 2-like and regulatory properties. Front Immunol. (2024) 15:1400550. doi: 10.3389/fimmu.2024.1400550, PMID: 38835756 PMC11148280

[97] Karwig L Moore PF Alber G Eschke M . Distinct characteristics of unique immunoregulatory canine non-conventional TCRαβpos CD4negCD8αneg double-negative T cell subpopulations. Front Immunol. (2024) 15:1439213. doi: 10.3389/fimmu.2024.1439213, PMID: 39185407 PMC11341405

[98] Rabiger FV Rothe K von Buttlar H Bismarck D Büttner M Moore PF . Distinct features of canine non-conventional CD4-CD8α- double-negative TCRαβ+ vs. TCRγδ+ T Cells Front Immunol. (2019) 10:2748. doi: 10.3389/fimmu.2019.02748, PMID: 31824515 PMC6883510

[99] Hakanen E Lehtimäki J Salmela E Tiira K Anturaniemi J Hielm-Björkman A . Urban environment predisposes dogs and their owners to allergic symptoms. Sci Rep. (2018) 8:1585. doi: 10.1038/s41598-018-19953-3, PMID: 29371634 PMC5785484

[100] Temizkan MC Sonmez G . Are owned dogs or stray dogs more prepared to diseases? A comparative study of immune system gene expression of perforin and granzymes. Acta Vet Hung. (2022) 70:24–9. doi: 10.1556/004.2022.00005, PMID: 35238799

[101] Cardillo L Piegari G Iovane V Viscardi M Alfano F Cerrone A . Lifestyle as risk factor for infectious causes of death in young dogs: A retrospective study in southern Italy (2015-2017). Vet Med Int. (2020) 2020:6207297. doi: 10.1155/2020/6207297, PMID: 32566119 PMC7293748

[102] Choi JW Withers SS Chang H Spanier JA de la Trinidad VL Panesar H . Development of canine PD-1/PD-L1 specific monoclonal antibodies and amplification of canine T cell function. PloS One. (2020) 15:e0235518. doi: 10.1371/journal.pone.0235518, PMID: 32614928 PMC7332054

[103] Chiku VM Silva KLO de Almeida BFM Venturin GL Leal AAC de Martini CC . PD-1 function in apoptosis of T lymphocytes in canine visceral leishmaniasis. Immunobiology. (2016) 221:879–88. doi: 10.1016/j.imbio.2016.03.007, PMID: 27016050

[104] Ammons DT Harris RA Chow L Dow S . Characterization of canine tumor-infiltrating leukocyte transcriptomic signatures reveals conserved expression patterns with human osteosarcoma. Cancer Immunol Immunother. (2025) 74:105. doi: 10.1007/s00262-025-03950-3, PMID: 39932553 PMC11813853

[105] Kim M-C Borcherding N Song W-J Kolb R Zhang W . Leveraging single-cell transcriptomic data to uncover immune suppressive cancer cell subsets in triple-negative canine breast cancers. Front Vet Sci. (2024) 11:1434617. doi: 10.3389/fvets.2024.1434617, PMID: 39376916 PMC11457229

[106] Holder A Mirczuk SM Fowkes RC Palmer DB Aspinall R Catchpole B . Perturbation of the T cell receptor repertoire occurs with increasing age in dogs. Dev Comp Immunol. (2018) 79:150–7. doi: 10.1016/j.dci.2017.10.020, PMID: 29103899 PMC5711257

[107] Miyai S Hendawy AO Sato K . Gene expression profile of peripheral blood mononuclear cells in mild to moderate obesity in dogs. Veterinary Anim Sci. (2021) 13:100183. doi: 10.1016/j.vas.2021.100183, PMID: 34258471 PMC8251507

[108] Jaffey JA Su D Monasky R Hanratty B Flannery E Horman M . Effects of a whole food diet on immune function and inflammatory phenotype in healthy dogs: A randomized, open-labeled, cross-over clinical trial. Front Vet Sci. (2022) 9:898056. doi: 10.3389/fvets.2022.898056, PMID: 36082214 PMC9447376

[109] Backer LC Grindem CB Corbett WT Cullins L Hunter JL . Pet dogs as sentinels for environmental contamination. Sci Total Environ. (2001) 274:161–9. doi: 10.1016/s0048-9697(01)00740-9, PMID: 11453293

[110] Moore PF Rossitto PV Danilenko DM Wielenga JJ Raff RF Severns E . Monoclonal antibodies specific for canine CD4 and CD8 define functional T-lymphocyte subsets and high-density expression of CD4 by canine neutrophils. Tissue Antigens. (1992) 40:75–85. doi: 10.1111/j.1399-0039.1992.tb01963.x, PMID: 1412420

[111] Biswas P Mantelli B Sica A Malnati M Panzeri C Saccani A . Expression of CD4 on human peripheral blood neutrophils. Blood. (2003) 101:4452–6. doi: 10.1182/blood-2002-10-3056, PMID: 12531788

[112] Hamza FN Alekhmimi NA Matar DSB Barakzai A Al-Kattan K Heialy SA . Pulmonary neutrophil recruitment in response to inhaled LPS is impaired in CD4 deficient mice. Res Sq. (2022). doi: 10.21203/rs.3.rs-1796185/v1

[113] Vanhaezebrouck IF Bakhle KM Mendez-Valenzuela CR Lyle LT Konradt K Scarpelli ML . Single institution study of the immune landscape for canine oral melanoma based on transcriptome analysis of the primary tumor. Front Vet Sci. (2023) 10:1285909. doi: 10.3389/fvets.2023.1285909, PMID: 38260202 PMC10800815

[114] Gow DJ Jackson H Forsythe P Gow AG Mellanby RJ Hume DA . Measurement of serum macrophage migration inhibitory factor (MIF) and correlation with severity and pruritus scores in client owned dogs with atopic dermatitis. Vet Dermatol. (2019). doi: 10.1111/vde.12721, PMID: 30672038

[115] Galadima M Kotova I Schmidt R Pastor J Schröder C Rodríguez-Gil JE . Canine mammary neoplasia induces variations in the peripheral blood levels of CD20, CD45RA, and CD99. Int J Mol Sci. (2023) 24. doi: 10.3390/ijms24119222, PMID: 37298173 PMC10252927

[116] Gobbini E Hubert M Doffin A-C Eberhardt A Hermet L Li D . The spatial organization of cDC1 with CD8+ T cells is critical for the response to immune checkpoint inhibitors in patients with melanoma. Cancer Immunol Res. (2025) 13:517–26. doi: 10.1158/2326-6066.CIR-24-0421, PMID: 39774795

