# Correction: High-resolution single-cell RNA sequencing using canFam4 reveals novel immune subsets and checkpoint programs in healthy dogs

**DOI:** 10.3389/fimmu.2025.1773404

**Published:** 2026-01-06

**Authors:** Myung-Chul Kim, Taeeun Gu, Hyeewon Seo, Yewon Moon, Nicholas Borcherding, Ryan Kolb, Yubin Kim, Youngmin Yun, Woo-Jin Song, Chung-Young Lee, Hyun Je Kim, Weizhou Zhang

**Affiliations:** 1 Laboratory of Clinical Pathology, College of Veterinary Medicine, Kyungpook National University, Daegu, Republic of Korea; 2 Department of Biomedical Sciences, Seoul National University Graduate School, Seoul, Republic of Korea; 3 Department of Pathology & Immunology, Washington University School of Medicine in St. Louis, St. Louis, MO, United States; 4 Department of Pathology, Immunology and Laboratory Medicine, University of Florida, College of Medicine, Gainesville, FL, United States; 5 College of Veterinary Medicine, Jeju National University, Jeju, Republic of Korea; 6 Laboratory of Veterinary Internal Medicine, College of Veterinary Medicine, Jeju National University, Jeju, Republic of Korea; 7 Signiture Animal Medical Center, Seoul, Republic of Korea; 8 Department of Microbiology, School of Medicine, Kyungpook National University, Daegu, Republic of Korea; 9 Untreatable Infectious Disease Institute, Kyungpook National University, Daegu, Republic of Korea; 10 Genomic Medicine Institute, Seoul National University Medical Research Center, Seoul, Republic of Korea; 11 Department of Microbiology and Immunology, Seoul National University College of Medicine, Seoul, Republic of Korea; 12 Cancer Research Institute, Seoul National University College of Medicine, Seoul, Republic of Korea; 13 UF Health Cancer Center, University of Florida, Gainesville, FL, United States

**Keywords:** canFam4, circulating leukocytes, reference transcriptome, ScRNA-seq, translational model

Affiliation “Department of Biomedical Sciences, Seoul National University Graduate School, Seoul, Republic of Korea” and “Cancer Research Institute, Seoul National University College of Medicine, Seoul, Republic of Korea” were omitted for author “Hyun Je Kim”. These affiliations have now been added for author “Hyun Je Kim”.

There was a mistake in the **Funding** statement. An incorrect number was provided for “Basic Science Research Program through the National Research Foundation of Korea (NRF) funded by the Ministry of Education” supporting MK. The correct number is “RS-2023-00241779”. An incorrect number was provided for “Bio & Medical Technology Development Program of the NRF funded by the Korean government” supporting HK. The correct number is “RS-2022-NR067309”.

The original version of this article has been updated.

