# Supplementary material for: High-resolution single-cell RNA sequencing using canFam4 reveals novel immune subsets and checkpoint programs in healthy dogs
Source: Front Immunol. 2025 Dec 18;16:1680437. doi: 10.3389/fimmu.2025.1680437 (PMC12756145; doi:10.3389/fimmu.2025.1680437)
Supplement: Supplementary file 1 [file DataSheet1.pdf]

Supplementary Material

High-resolution single-cell RNA sequencing using canFam4 reveals novel immune subsets and checkpoint programs in healthy dogs

Myung-Chul Kim, Taeeun Gu, Hyeewon Seo, Yewon Moon, Nicholas Borcherding, Ryan Kolb, Yubin Kim, Youngmin Yun ,Woo-Jin Song, Chung-Young Lee, Hyun Je Kim, Weizhou Zhang

*** Correspondence:** Myung-Chul Kim: mck@knu.ac.kr

1 Supplementary Data

Supplementary Material should be uploaded separately on submission. Please include any supplementary data, figures and/or tables.

Supplementary material is not typeset so please ensure that all information is clearly presented, the appropriate caption is included in the file and not in the manuscript, and that the style conforms to the rest of the article.

2 Supplementary Figures and Tables

For more information on Supplementary Material and for details on the different file types accepted, please see here.

2.1 Supplementary Figure

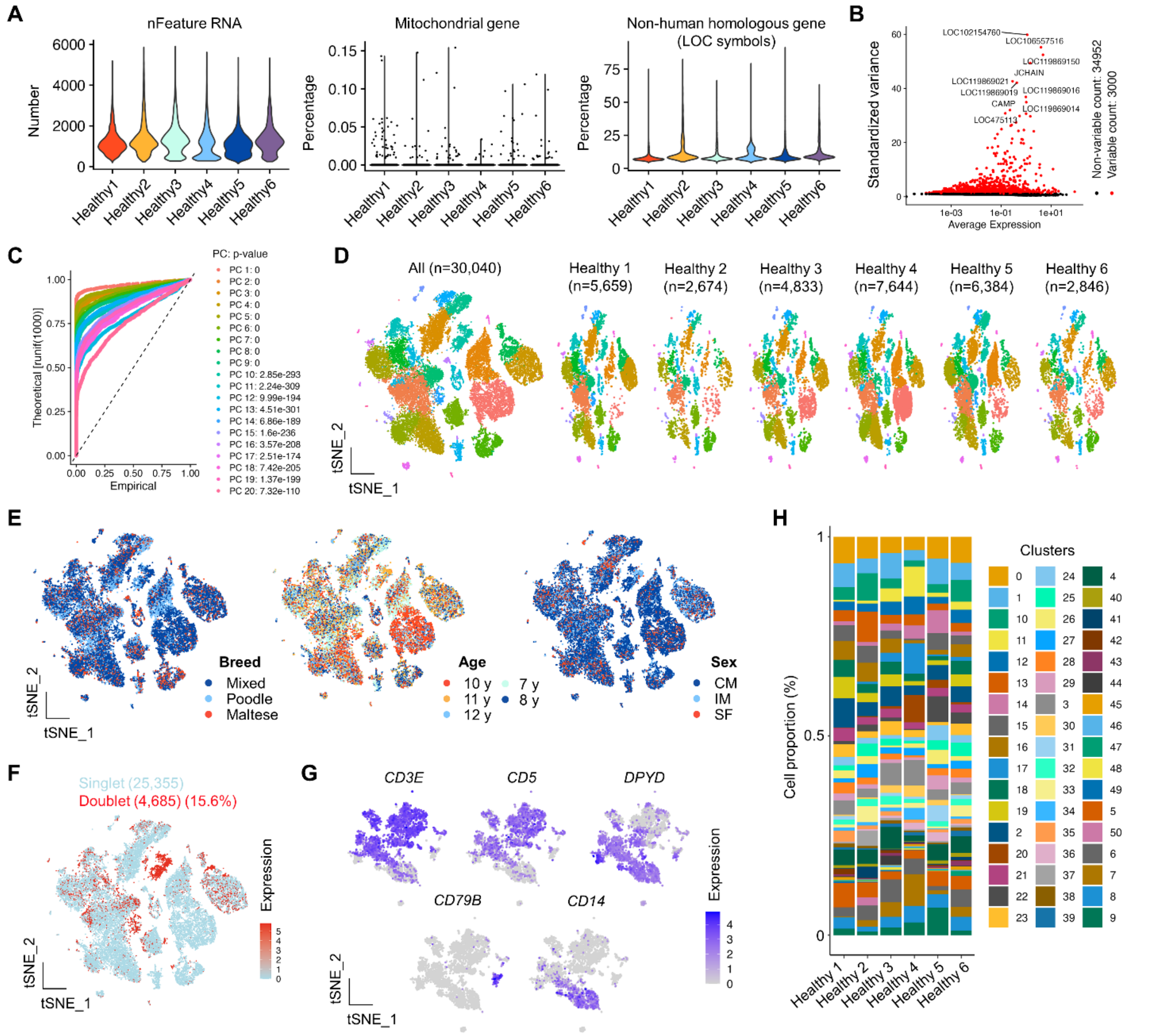

Supplementary Figure 1. Preprocessing and quality control of the scRNA-seq data in Seurat. (A) Violin plots of the distribution of unique genes detected per cell (nFeature_RNA), the percentage of mitochondrial reads, and the percentage of non-human homologous genes across biological replicates. (B) Selection of 3,000 highly variable features exhibiting high cell-to-cell variation within the integrated dataset. (C) PC analysis enriched for informative features (low p-values) is visualized using a JackStraw plot. (D) tSNE plot of the immune cells from each biological replicate, annotated with cell counts. (E) The tSNE distribution of immune cells by breed, age, and sex. No notable differences were observed across these groups, and minor visual variations were not biologically or statistically meaningful given the small cohort size (n = 6). (F) Cell proportions across clusters and biological replicates. (G) Distribution and proportion of potential doublets detected in the integrated Seurat object. (H) Subclustering of predicted doublets, revealing co-expression of T, B, and myeloid lineage markers on the tSNE feature plot. Abbreviations: CM, castrated male; IM, intact male; SF, spayed female; PC, principal component.

.

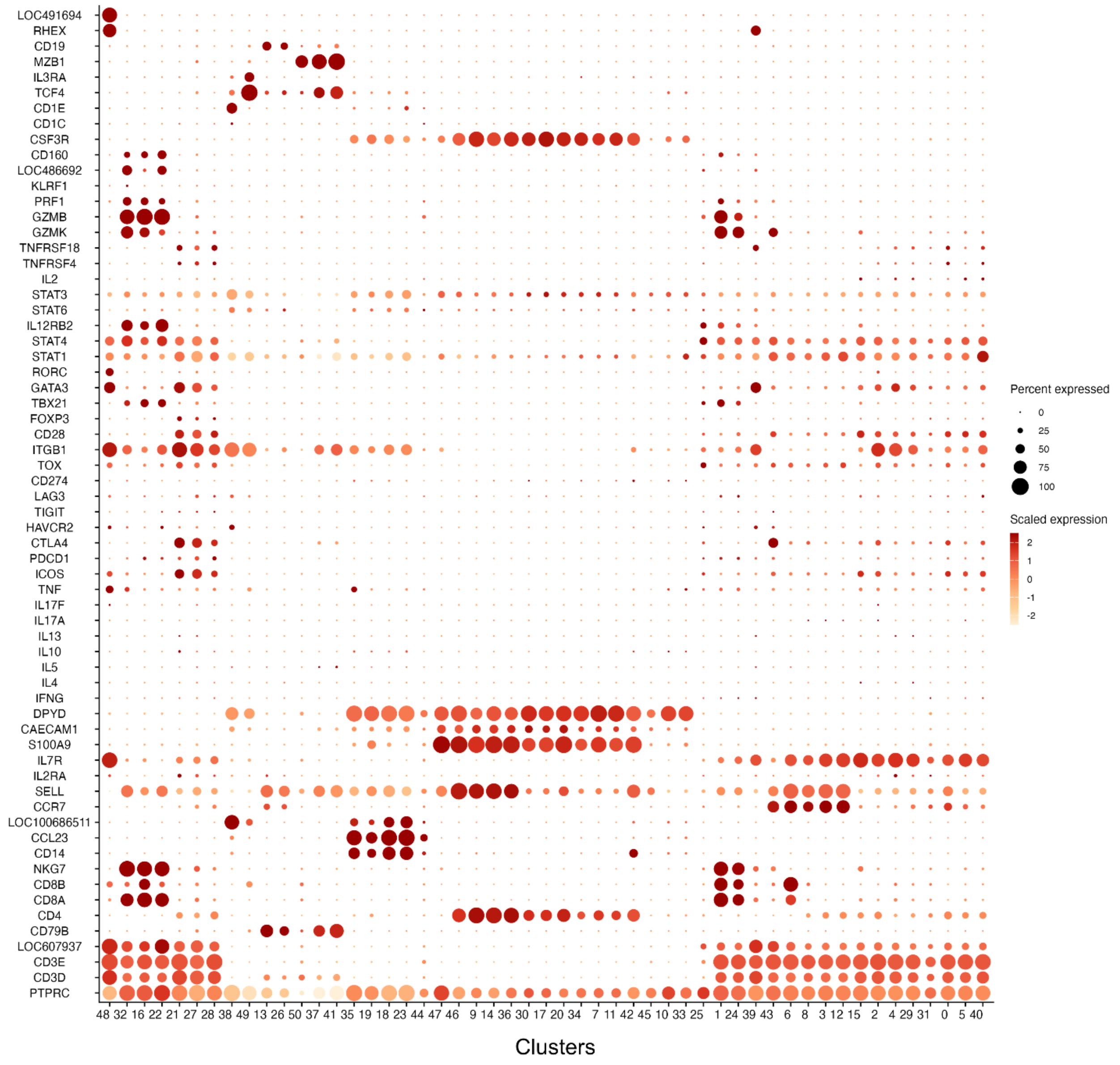

Supplementary Figure 2. Canonical marker expression profiles used to define distinct single-cell immune clusters. Dot plot of the representative canonical markers used to annotate transcriptionally and functionally distinct immune subsets within the integrated scRNA-seq dataset.

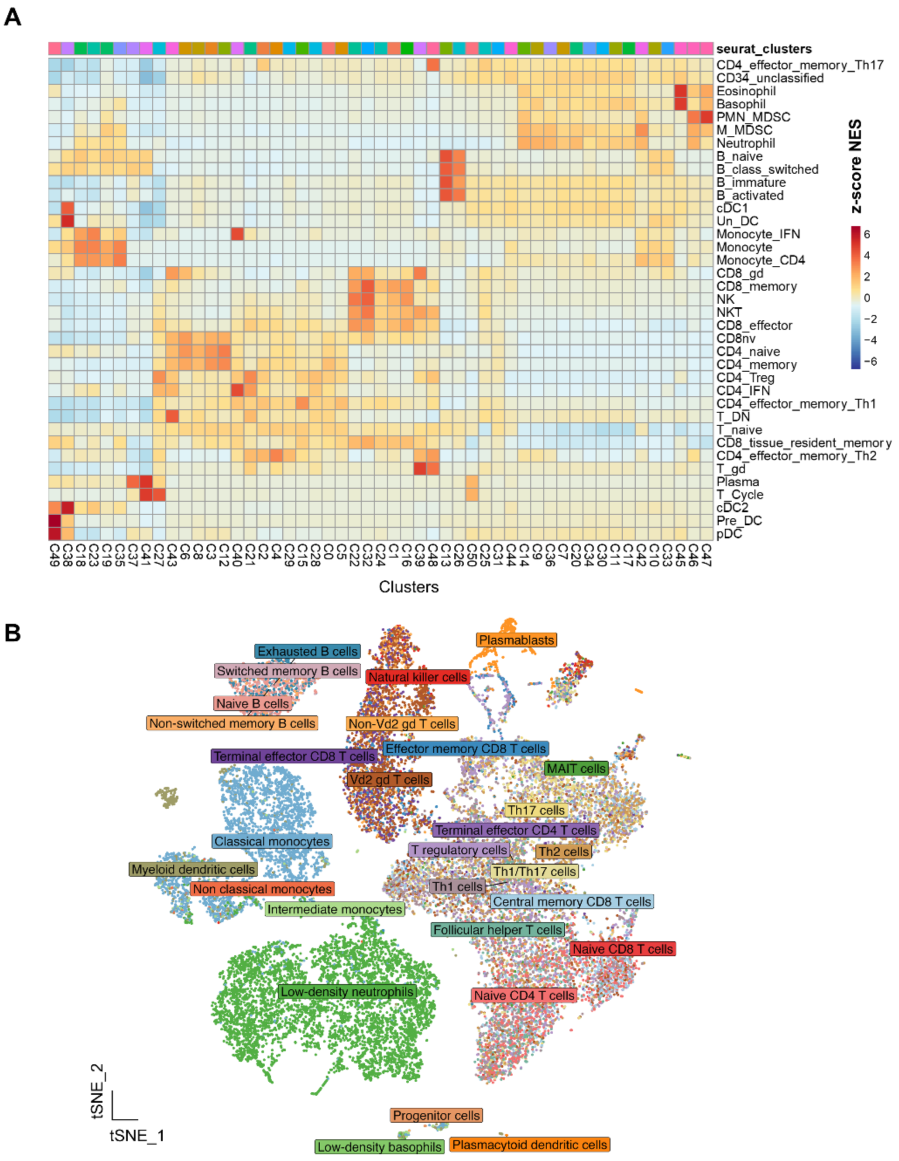

Supplementary Figure 3. Unbiased cell type recognition using reference-based annotation tools. (A) Heatmap of the cell type predictions using the escape R package, based on canine immune cell gene signatures derived from previous studies (8,22). (B) A tSNE plot of the cell type annotations performed using the SingleR R package with the Monaco Immune Cell Data as the reference dataset.

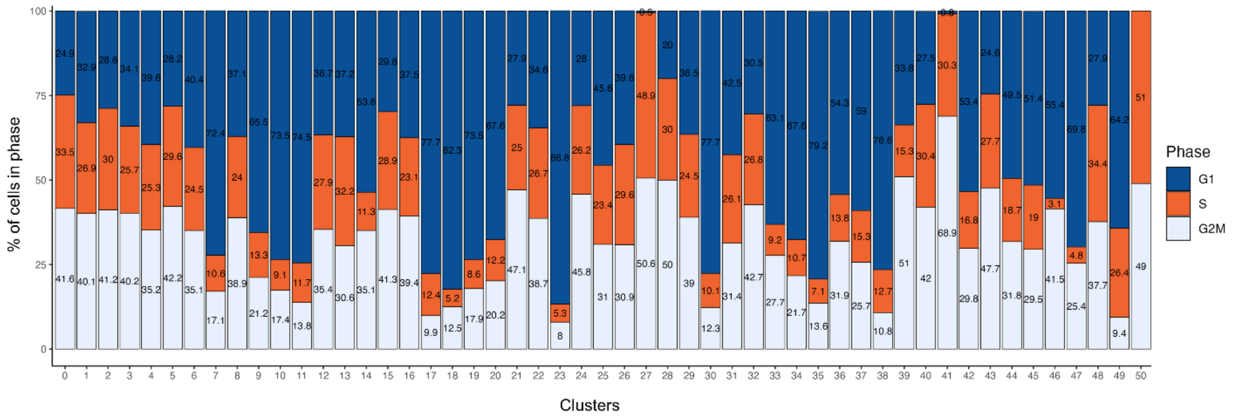

Supplementary Figure 4. Cell cycle analysis of immune cell subsets. Cell cycle phase distribution (G1, S, G2/M) is shown across immune cell subsets within the integrated scRNA-seq dataset.

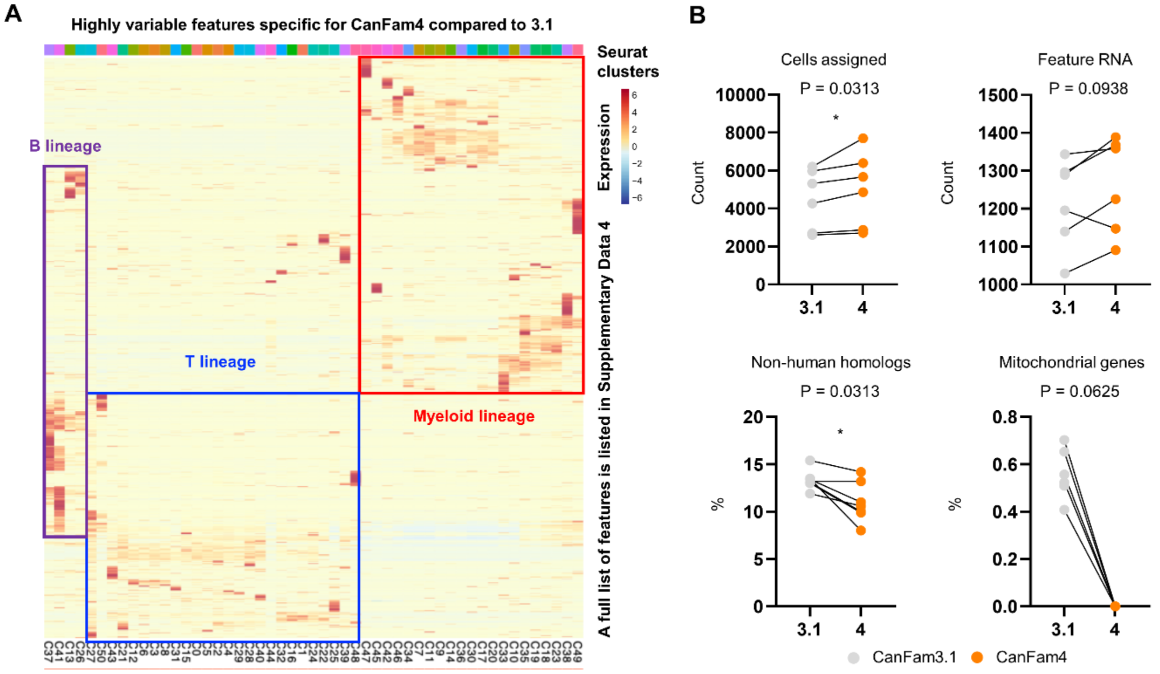

Supplementary Figure 5. Preprocessing and comparative analysis of scRNA-seq data using canFam3.1 and canFam4 reference genomes. (A) Quality control metrics across the two canine genome references, including the number of cells per sample, number of unique genes detected per cell, percentage of non-human homologous genes, and percentage of mitochondrial reads. Each dot represents a biological replicate. (B) A tSNE plot of the 48 clusters identified from the integrated dataset aligned to canFam3.1. (C) The tSNE density plots of the the distribution of CD8^{+} T cell subsets between canFam3.1 and canFam4 references. (D) Heatmap of the representative genes among the top 10 DEGs used to define each major immune lineage. Cell counts per lineage are indicated below the heatmap. A full list of highly variable features specific to canFam4 compared to canFam3.1 is provided in **Supplementary Data 4**.

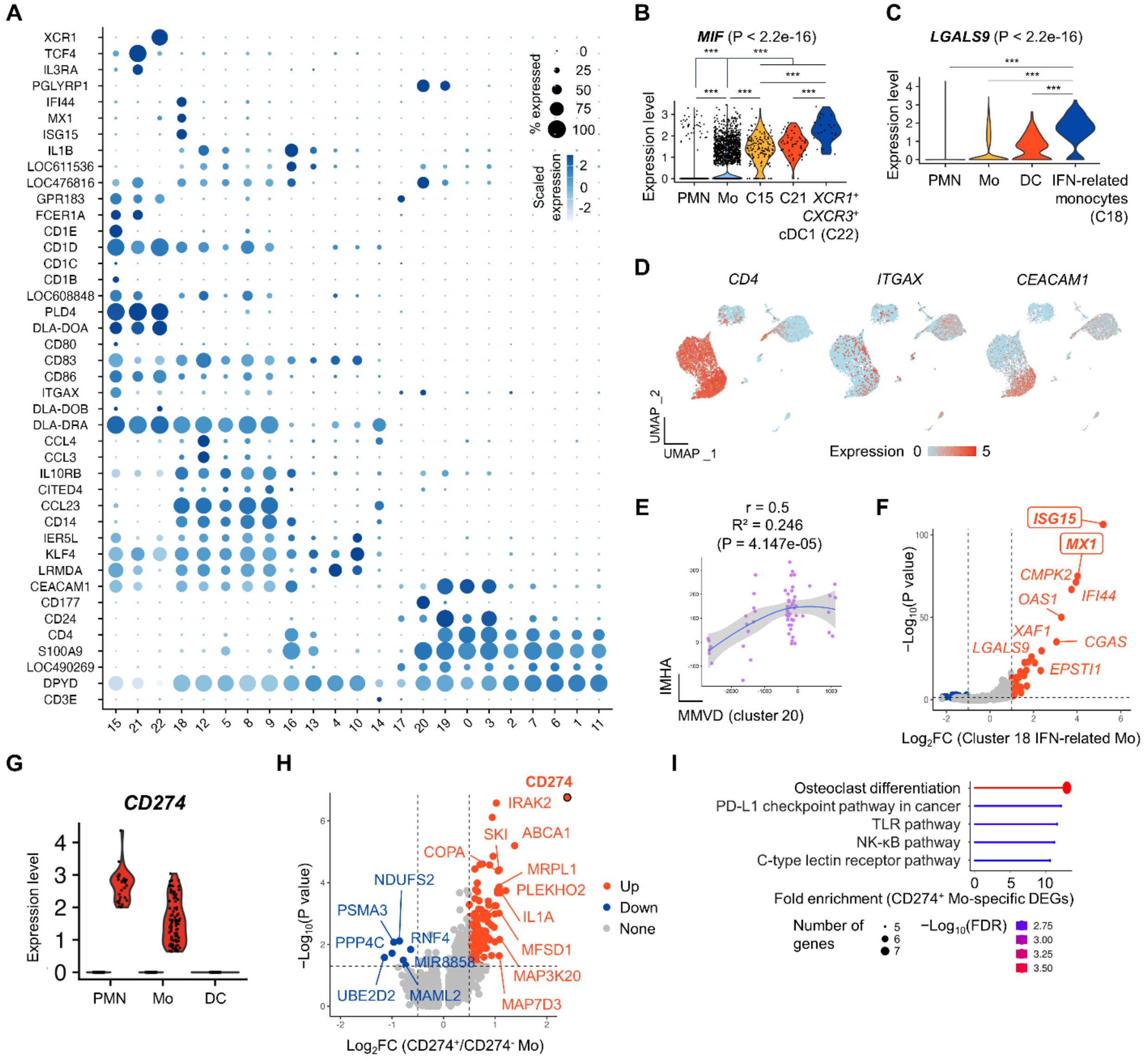

Supplementary Figure 6. Identification and characterization of myeloid subpopulations by scRNA-seq. (A) Dot plot of the canonical marker expression profiles used to define transcriptionally and functionally distinct myeloid cell subsets. Cluster annotations correspond to those in **Figure 2**. Violin plots of the expression pattern of *MIF* (B) and *LGALS9* (C) on myeloid subsets. (D) Feature plots of the expression patterns of *CD4*, *ITGAX*, and *CEACAM1* in myeloid cells. (E) Scatter plot of the correlation between MMVD and IMHA signatures in cluster 20. (F) Volcano plot of the representative DEGs in IFN-related monocytes compared to other monocyte subsets. (G) Violin plot of the segregation of myeloid subsets based on *CD274* expression. (H) Volcano plot of the representative DEGs in *CD274*$^{+}$ monocytes relative to *CD274*$^{-}$ monocytes. *CD274* is highlighted with a $\log_2$FC of 12.8 and **P** = 0. (I) GO analysis of *CD274*$^{+}$ monocyte-specific DEGs reveals enrichment in biological processes related to the PD-L1 checkpoint pathway in cancer. Abbreviations: FC, fold change; DEG, differentially expressed genes; GO, gene ontology.

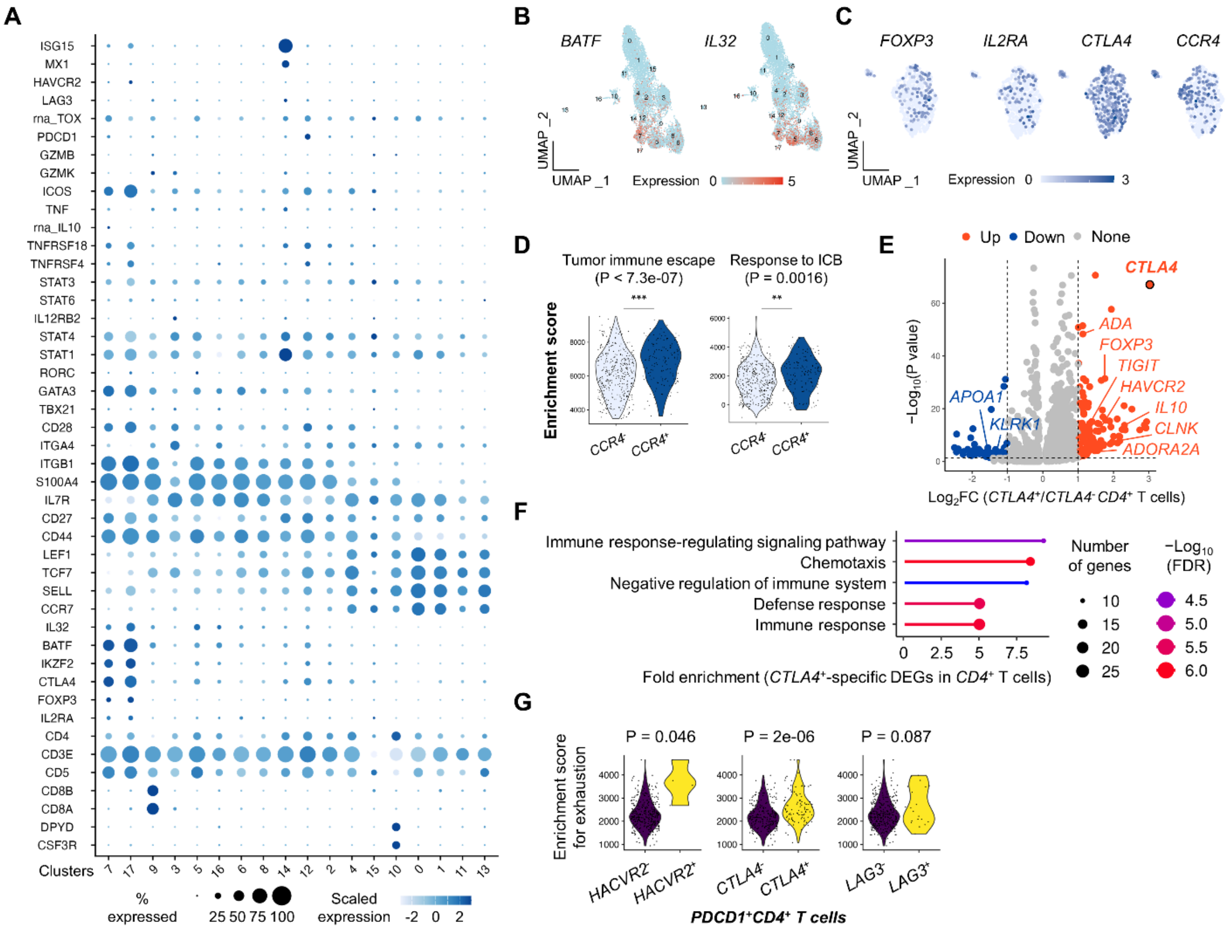

Supplementary Figure 7. Identification and characterization of *CD4*$^+$ T subpopulations by scRNA-seq. (A) Dot plot of the canonical markers used to identify functionally distinct CD4$^+$ T cell subsets. (B) Feature plots of the expression patterns of *BATF* and *IL32*. (C) Feature plot of the expression patterns of *FOXP3*, *IL2RA*, *CTLA4*, and *CCR4* in Tregs subclustered from clusters 7 and 17. (D) Violin plots of the increased enrichment of gene sets associated with tumor immune escape and response to immune checkpoint blockade in *CCR4*$^+$ Tregs. (E) Volcano plot of the representative *CTLA4*-specific DEGs that belong to the gene set of negative regulation of immune system. (F) GO analysis of *CTLA4*$^+$*CD4*$^+$ T-specific DEGs. (G) Violin plots of the increased enrichment of exhaustion gene set in *HAVCR2*$^+$ and *CTLA4*$^+$*PDCD1*$^+$*CD4*$^+$ T cells. Statistical significance and P values was determined by comparing two groups of interest using the non-parametric Wilcoxon rank-sum test in the ggpubr R package.

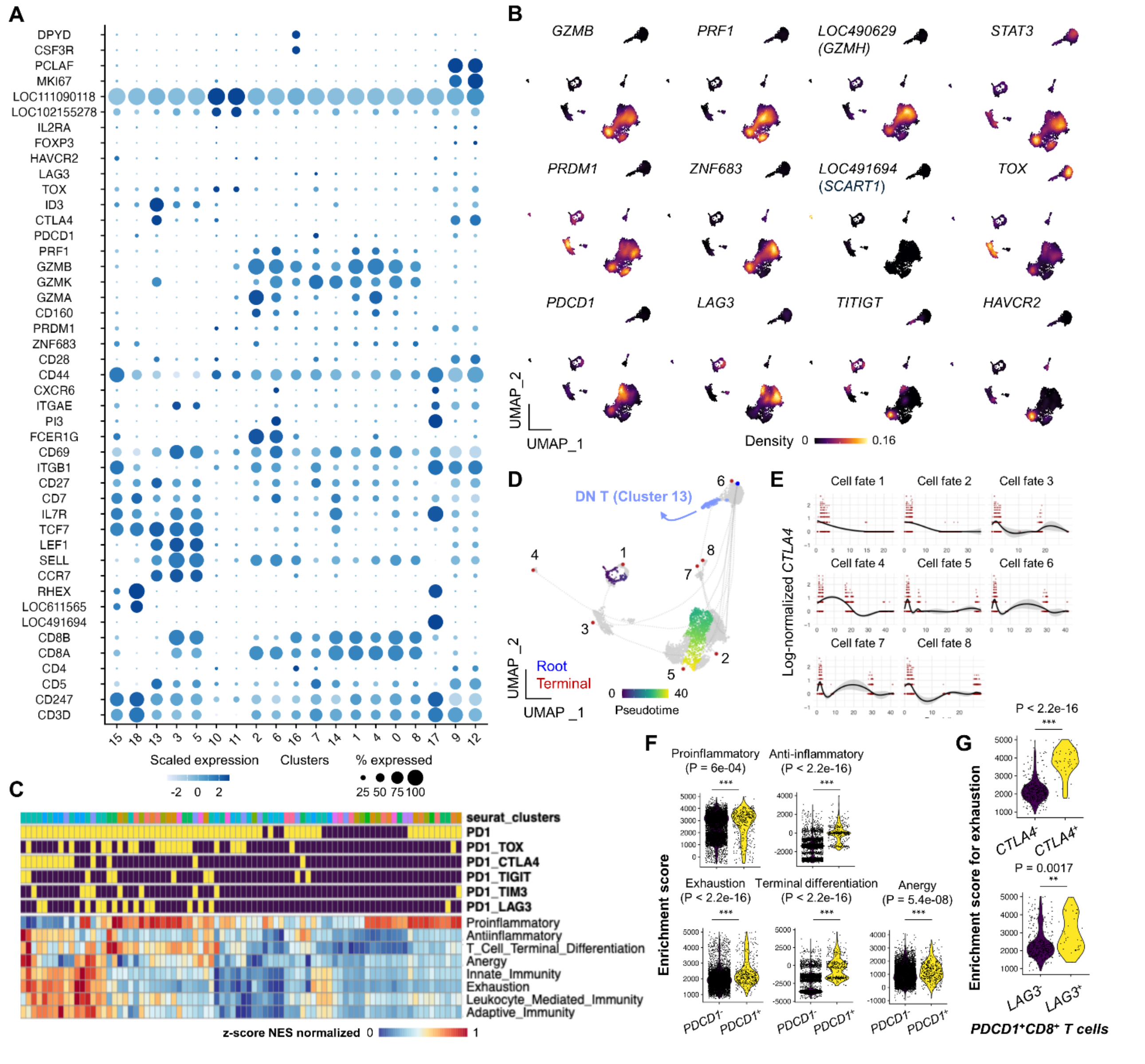

Supplementary Figure 8. Identification and characterization of *CD8*$^{+}$ T and miscellaneous subpopulations by scRNA-seq. (A) Canonical marker expression profile used to define distinct CD8^{+} and miscellaneous T cell clusters. Representative canonical markers are shown in the dot plot. (B) Feature plots of the expression of *CD247* (*CD3Z*), *CD4*, and *CD8A*. (C) GSEA data of the immune-associated pathway enrichment in *CD8*$^{+}$ T cell clusters (clusters 0, 1, 2, 3, 4, 5, 6, 7, 8, and 14) with IC expression. (D) Slingshot-based pseudotime trajectory of *CD8*$^{+}$ cells projected onto UMAP space. Red and blue dots refers to terminal and root nodes, respectively. Cells with gray indicate unassigned pseudotime. (E) *CTLA4* expression dynamics along eight terminal lineages or cell fates. Black lines represent smoothed local regression, and red dots indicate individual cell measurements. (F) Violin plots of the enrichment pattern of immune-associated gene sets in *CD8*$^{+}$ T cells with or without *PDCD1*. (G) Violin plots of the increased enrichment of exhaustion gene set in *CTLA4*$^{+}$ and *LAG3*$^{+}$ *PDCD1*$^{+}$*CD8*$^{+}$ T cells.

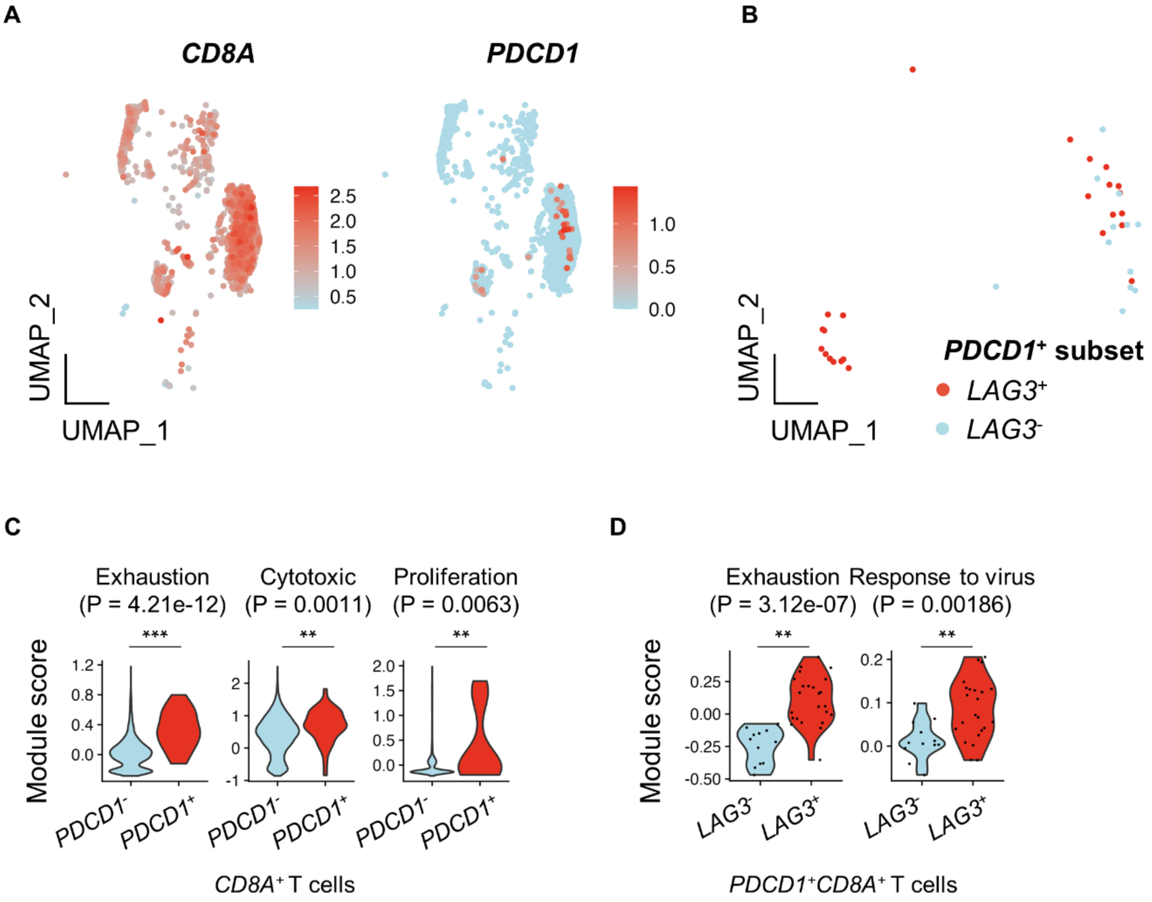

Supplementary Figure 9. Validation of transcriptional programs in tumor-infiltrating CD8⁺ T cells from canine osteosarcoma scRNA-seq datasets. Feature plots of the expression of (**A**) *CD8A* and *PDCD1*, and (**B**) *LAG3* in canine osteosarcoma-infiltrating *CD8*⁺ T and *PDCD1*⁺*CD8*⁺ T cells. (**C**) Module score analysis of exhaustion, cytotoxic, and proliferation programs between *PDCD1*⁺ and *PDCD1*⁻*CD8*⁺ T cell subsets. (**D**) Module score analysis of exhaustion and response to virus programs in *LAG3*⁺ versus *LAG3*⁻*PDCD1*⁺*CD8*⁺ T cells. Statistical significance and P values was determined by comparing two groups of interest using the non-parametric Wilcoxon rank-sum test in the ggpubr R package.

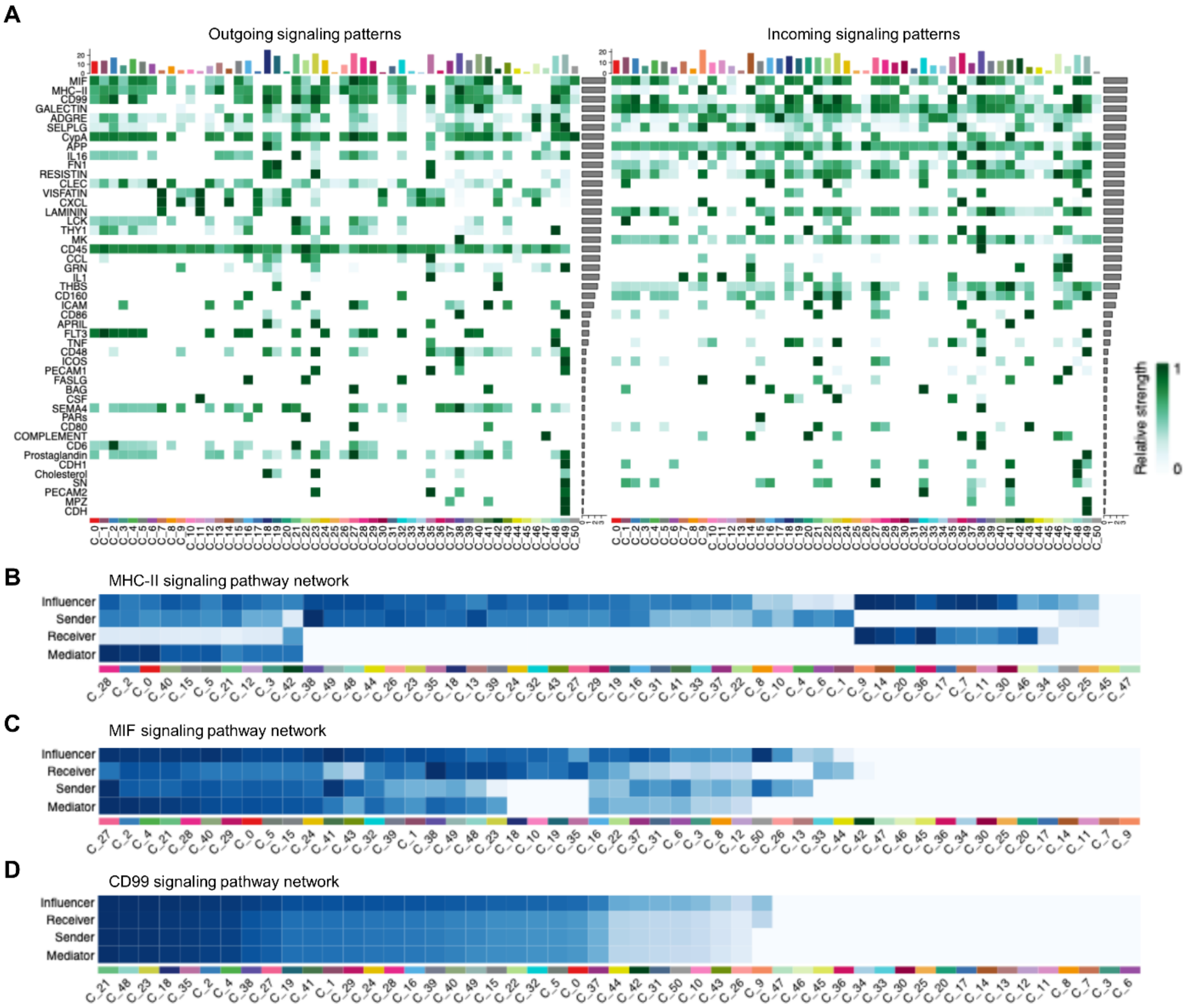

Supplementary Figure 10. Inference of immune cell communication network by CellChat analysis. (A) Overall outgoing and incoming signaling patterns of significant pathways across clusters. Bar plots represent the total interaction strength for each sending (column) and receiving (row) cell type. (B–D) Network centrality analysis identifies the dominant signal senders, receivers, mediators, and influencers in each indicated signaling pathway.

.

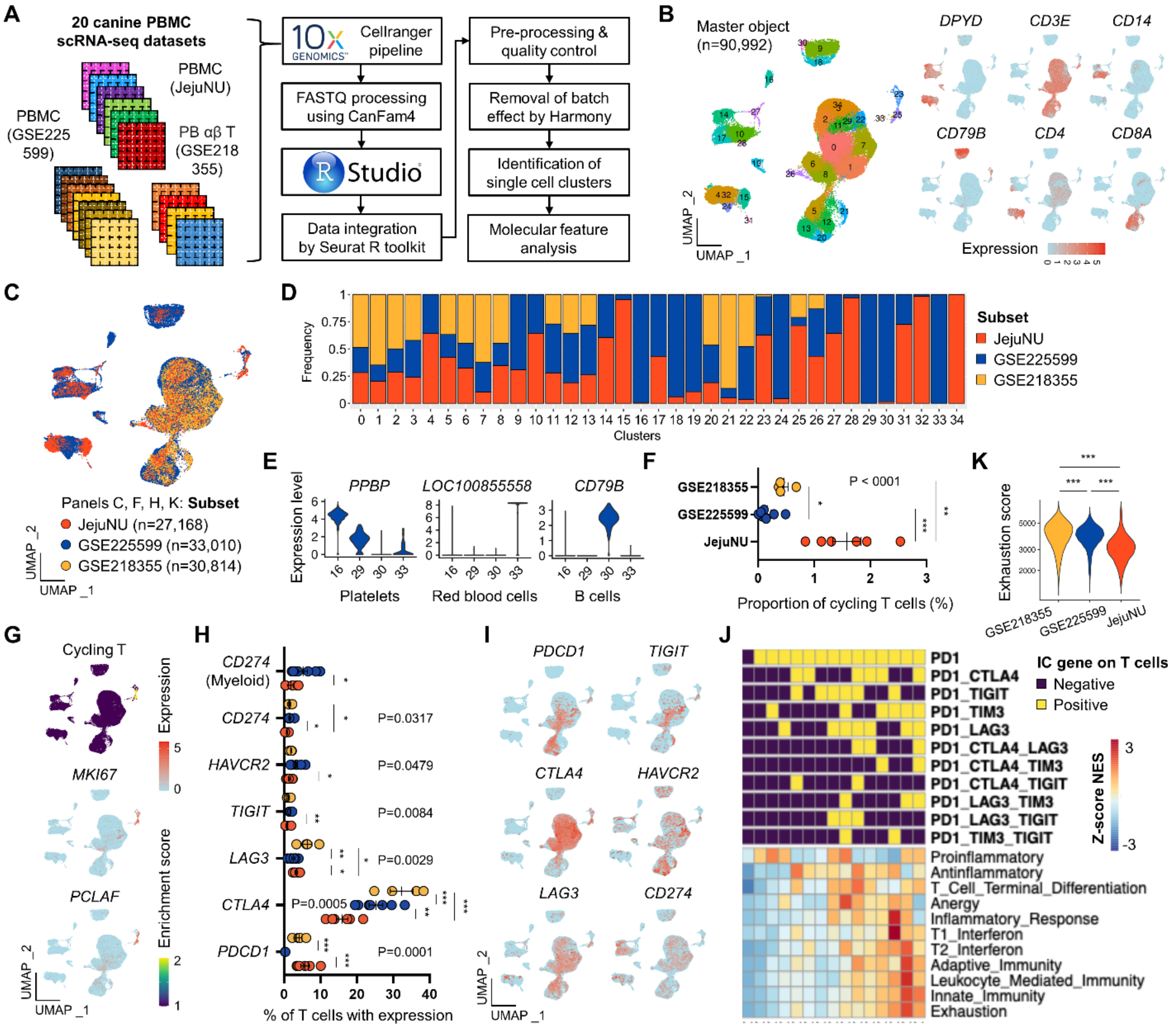

Supplementary Figure 11. Integrated scRNA-seq analysis reveals cohort-specific immune features in healthy dogs. (A) Study scheme showing the overview of 20 healthy canine PBMC scRNA-seq datasets used in this study. (B) UMAP plot of the integrated Seurat dataset comprising 90,992 single cells classified into 35 clusters. Canonical immune markers used to identify the major subsets are indicated. (C) UMAP plot of the cell distribution across different canine cohorts. (D) Bar plot of the relative abundance of immune subsets across datasets, (E) Violin plots of the representative genes enriched in specific subsets identified in the GSE225599 dataset. (F) Proportional analysis of a study-specific cycling T subset (cluster 25). (G) Feature plot of the T cell cycle gene signatures and proliferation-related genes in cycling T cells. (H) Comparative analysis of IC gene–expressing T cell proportions, showing cohort-specific presence of *CTLA4*$^{+}$ and *LAG3*$^{+}$ cells. (I) Dimensional plot of the spatial distribution of T cells expressing IC genes (*CTLA4*, *LAG3*), visualized in the front layer. (J) GSEA results indicate that immune-related pathways are enriched in T cells expressing IC genes. The *p*-values were obtained using one-way ANOVA via the ggpubr R package and Wilcoxon rank-sum tests. * $p < 0.05$, ** $p < 0.01$, and *** $p < 0.001$.

Supplementary Table Legends

Supplementary Data 1. Demographic characteristics of enrolled dogs.

Supplementary Data 2. Complete blood count and serum biochemical test results.

Supplementary Data 3. Summary of scRNA-seq library construction and multiplexing statistics.

Supplementary Data 4. Representative canonical and canFam4-annotated genes used to define major immune subsets in healthy dogs.

Supplementary Data 5. Highly variable gene features identified for canFam3.1 and canFam4 genomes.

Supplementary Data 6. Top 20 cluster-defining genes for each myeloid cell subset.

Supplementary Data 7. *CD274*-specific DEGs in myeloid cells.

Supplementary Data 8. GO terms enriched from genes listed in Supplementary Data 7.

Supplementary Data 9. Top 20 cluster-defining genes for each $CD4^+$ T cell subset.

Supplementary Data 10. *CCR4*-specific genes identified in Tregs.

Supplementary Data 11. GO terms enriched from genes listed in Supplementary Data 9.

Supplementary Data 12. *PDCD1*-specific genes in $CD4^+$ T cells.

Supplementary Data 13. *CTLA4*-specific genes in $CD4^+$ T cells.

Supplementary Data 14. DEGs associated with co-inhibitory receptors in $PDCD1^+CD4^+$ T cells.

Supplementary Data 15. Top 10 cluster-defining genes for each $CD8^+$ and miscellaneous T cell subset.

Supplementary Data 16. *PDCD1*-specific genes in $CD8^+$ T cells.

Supplementary Data 17. GO terms enriched from genes listed in Supplementary Data 15.

Supplementary Data 18. DEGs associated with co-inhibitory receptors in $PDCD1^+CD8^+$ T cells.

Supplementary Data 19. Top 20 cluster-defining genes in the integrated scRNA-seq dataset.
